# Transcriptome profiling of *Camelina sativa* to identify genes involved in triacylglycerol biosynthesis and accumulation in the developing seeds

**DOI:** 10.1186/s13068-016-0555-5

**Published:** 2016-07-04

**Authors:** Hesham M. Abdullah, Parisa Akbari, Bibin Paulose, Danny Schnell, Weipeng Qi, Yeonhwa Park, Ashwani Pareek, Om Parkash Dhankher

**Affiliations:** Stockbridge School of Agriculture, University of Massachusetts Amherst, Amherst, MA 01003 USA; Biotechnology Department, Faculty of Agriculture, Al-Azhar University, Cairo, 11651 Egypt; Department of Plant Biology, Michigan State University, East Lansing, MI 48824 USA; Department of Food Science, University of Massachusetts Amherst, Amherst, MA 01003 USA; Stress Physiology and Molecular Biology Laboratory, School of Life Science, Jawaharlal Nehru University, New Delhi, 100067 India

**Keywords:** *Camelina sativa*, Triacylglycerol biosynthesis, Transcriptome profiling, Lipid metabolism, Fatty-acid profiling

## Abstract

**Background:**

*Camelina sativa* is an emerging dedicated oilseed crop designed for biofuel and biodiesel applications as well as a source for edible and general-purpose oils. Such valuable oilseed crop is subjected to plant breeding programs and is suggested for large-scale production of better seed and oil quality. To accomplish this objective and to further enhance its oil content, a better understanding of lipid metabolism at the molecular level in this plant is critical. Here, we applied tissue transcriptomics and lipid composition analysis to identify and profile the genes and gene networks associated with triacylglycerol (TAG) biosynthesis, and to investigate how those genes are interacting to determine the quantity and quality of Camelina oil during seed development.

**Results:**

Our Camelina transcriptome data analysis revealed an approximate of 57,854 and 57,973 genes actively expressing in developing seeds (RPKM ≥ 0.1) at 10–15 (Cs-14) and 16–21 (Cs-21) days after flowering (DAF), respectively. Of these, 7932 genes showed temporal and differential gene expression during the seed development (log2 fold change ≥1.5 or ≤−1.5; *P* ≤ 0.05). The differentially expressed genes (DEGs) were annotated and were found to be involved in distinct functional categories and metabolic pathways. Furthermore, performing quantitative real-time PCR for selected candidate genes associated with TAG biosynthesis validated RNA-seq data. Our results showed strong positive correlations between the expression abundance measured using both qPCR and RNA-Seq technologies. Furthermore, the analysis of fatty-acid content and composition revealed major changes throughout seed development, with the amount of oil accumulate rapidly at early mid seed development stages (from 16–28 DAF onwards), while no important changes were observed in the fatty-acid profile between seeds at 28 DAF and mature seeds.

**Conclusions:**

This study is highly useful for understanding the regulation of TAG biosynthesis and identifying the rate-limiting steps in TAG pathways at seed development stages, providing a precise selection of candidate genes for developing Camelina varieties with improved seed and oil yields.

**Electronic supplementary material:**

The online version of this article (doi:10.1186/s13068-016-0555-5) contains supplementary material, which is available to authorized users.

## Background

Triacylglycerols (TAGs) are types of neutral lipids with considerable importance for dietetic, industrial, cosmetic, and pharmaceutical applications, as well as alternative energy sources for biofuel and biodiesel applications [1]. The function and value of different plant oils are derived from the fatty-acids composition of TAGs, thus determining the market values for those oils. Kennedy first formulated the TAG biosynthesis pathway in 1961 [2], and since then, the synthesis of glycerolipids has become one of the hallmarks of lipid biochemistry. In the Kennedy pathway, TAG synthesis is controlled by sequential acylation for glycerol-3-phosphate (G3P) and lysophosphatidic acid (LPA), following by dephosphorylation of phosphatidic acid (PA) to generate diacylglycerol (DAG). The latter is utilized by different classes of enzymes to ultimately generate TAGs that accumulate in the oil bodies of the mature seeds [2].

To identify the rate-limiting steps in TAG biosynthesis, several studies have been conducted, and many genes contributing to TAGs turnover have been characterized. Among these genes, diacylglycerol acyltransferases (*DGATs*) and phospholipid: diacylglycerol acyltransferases (*PDATs*) were shown to catalyze the final acylation steps during TAG synthesis, showing a substantial influence on the content and composition of some plant seed oils [3], and, thus, were suggested as rate-limiting enzymes in TAG biosynthesis. On the other hand, mutations in the glycerol-3-phosphate acyltransferase (*GPAT*) gene isoforms have significantly influenced the rate of TAG accumulation, and thus also suggested to be one of the limiting factors in TAG synthesis [4]. However, it remains poorly understood in plants as how these genes control TAG synthesis and how they are regulated as the seeds develop.

To reveal the role of individual genes in the synthesis and accumulation of TAG molecules into plant seeds, oilseed crops have attracted considerable interest. Among those crops, *Camelina sativa* (also known as false flax; referred hereafter as Camelina) has attracted much interest due to its unique seeds and oil qualities. The oil content of Camelina ranges from 30–40 % on a dry matter basis, and its oil is considered an excellent source for Omega 3 fatty acids, because of high alpha-linolenic acid (C18 ω3, 30–35 % of the total fatty acids) contents [5]. Camelina oil also contains high levels of vitamin E and antioxidants, which provide long shelf life for Camelina oil-containing products. Recently, Camelina has been engineered for the production of fish oil-like levels of DHA (docosahexaenoic acid, 22:6ω3) and EPA (eicosapentaenoic acid, 20:5ω3), which have various health benefits [6]. Beside the better oil qualities of Camelina, it also exhibits several positive agronomic attributes.

Camelina produces high seed yields, range from 800–1200 lbs/acre, in a relatively short growing season (85–100 days). Furthermore, many Camelina varieties grown in different climate areas appear to be cold and drought tolerant [6], and some of these varieties can grow and produce reasonable seed yields in high saline and low input soils [5]. Furthermore, Camelina is easy to transform using the floral-dip method [7], which facilitates the introduction of new useful traits into Camelina. These attributes make Camelina an ideal crop for large-scale production of seed oil as an alternative and environmentally friendly source for high-quality biodiesel [8]. Recently, several attempts have been made to identify and quantify the whole transcriptome of Camelina, focusing on the traits involved in stress response and disease resistance [9], lipid metabolism, and protein meal quality [10, 11]. However, from these studies, it is not clear how the TAG biosynthesis pathway genes are regulated and how they affect the oil composition and quantity during seed developmental stages. The availability of the Camelina reference genome released in 2014 [12] prompted us to perform transcriptome profiling for both developing seeds and leaf tissues using a genome-guided assembly approach, unlike the previous studies [9–11], in which de novo assembly was used for mapping.

Here, we used deep RNA sequencing to generate a high-resolution transcriptome map for *C. sativa* cultivar ‘Suneson’, during seed development with deep coverage of expressed sequences and reliable quantitative data. We report here a comprehensive analysis of Camelina transcriptome profiling at the stage-specific gene expression during the early (10–15 DAF, days after flowering) and early mid (16–21 DAF) stages of seed development. This facilitates the identification of differentially expressed genes (DEGs) within developing seeds, assessment of the gene expression patterns, and understanding the regulation of the genes involved in fatty-acid biosynthesis and TAG turnover, as well as the identification of functional categories to which these genes may belong. The approach used here is helpful to understand the molecular basis of TAG synthesis, identifying the rate-limiting genes/gene networks during seed development, and to select the ideal candidate genes to create improved varieties, producing better oil yield and composition.

## Results and discussion

### Genes associated with TAG biosynthesis are developmentally regulated

**Fig. 1:**
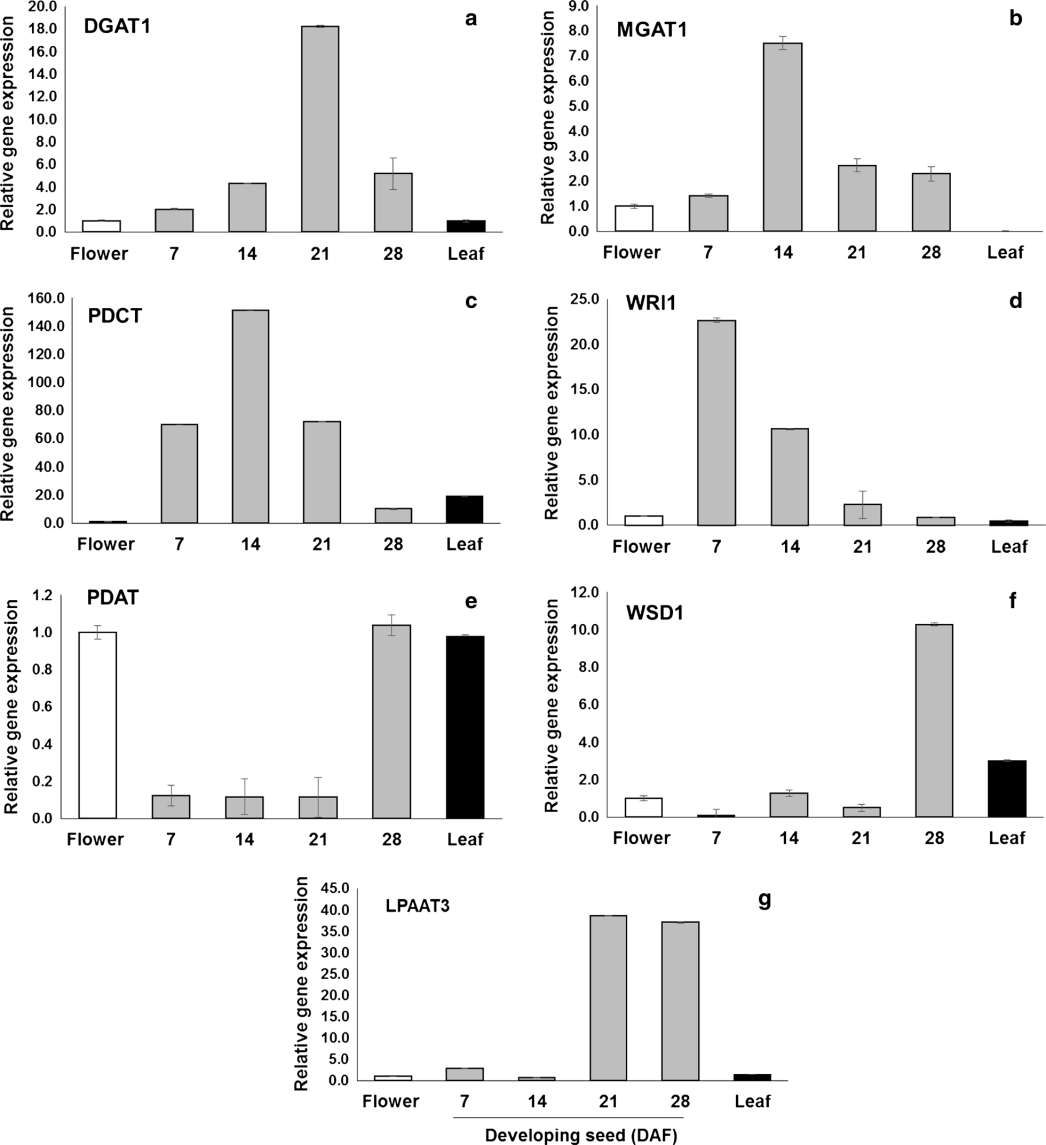
Expression profiling of selected Camelina TAG-associated genes. DGAT1 (**a**), MGAT1 (**b**), PDCT (**c**), WRl1 (**d**), PDAT1 (**e**), WSD1 (**f**), and LPAT3 (**g**) examined by quantitative real-time PCR in different camelina tissues; developing seeds, leaves, and flower tissues. Gene expression levels were normalized with respect to the internal control Actin2. *Data bars* represent the mean ± SE level of relative transcript abundance of three replicates. DAF days after flowering. Abbreviated names for the genes are described in Additional file 1: Table S1

Quantitative RT-PCR was performed to examine the steady-state mRNA levels of seven selected candidate genes, known to contribute to TAG metabolism, from Camelina seeds (Fig. 1). The flower samples were used as calibrators to represent the baseline of gene expression to monitor the change in the expression levels for the examined genes in developing Camelina seeds. As shown in Fig. 1, *DGAT1*, monoacylglycerol acyltransferase (*MGAT1*), phosphatidylcholine: diacylglycerol cholinephosphotransferase (*PDCT*), lysophosphatidyl acyltransferase 3 (*LPAT3*), and Wrinkled 1 (*WRl1*) genes showed seed-specific expression patterns with a relative maximum expression ratio (seed-to-flower ratio) up to ~38-fold and 150-fold in the case of *LPAT3* and *PDCT,* respectively. On the other hand, *PDAT* and *WSD1* (Wax ester synthase/acyl-CoA:diacylglycerol acyltransferase) *genes* showed low background expression in seed tissues at 7, 14, and 21 DAF, but not in seeds at 28 DAF, while their expression was extremely high in flower and leaf tissues, respectively. During the Camelina seed development, the selected TAG genes displayed various temporal and enhanced expression patterns, such as bell-shaped (*DGAT1*, *MGAT1*, and *PDCT*) and flat-rise (*LPAT3*) patterns, whereas *WRl1* exhibited a specific declining pattern (Fig. 1). It has been reported that the oil accumulation period in Camelina seed is between 18 to 24 DAF [13]), which coincides with the expression patterns of TAG genes investigated here. The expression of *DGAT1* and *LPAT3* peaks at 21 DAF, which suggest their involvement in TAG assembly and accumulation, whereas, *PDCT* and *MGAT1* genes showed maximum expression at earlier stage (14 DAF), thus supporting the fact that these two genes may play roles in lipid metabolism during the period prior to oil deposition in seeds. Furthermore, the detection of the transcripts for both *PDAT* and *WSD1* genes in the non-seed tissues indicated their involvement in other lipid-related processes taking place in those tissues. The relatively high expression levels observed for *WSD1* gene in leaf tissues appear to correlate with its role in the synthesis of cuticular waxes, as reported previously in *Arabidopsis* leaf and stem tissues [14], indicating the probable wax synthase activity of Camelina *WSD1*. On the other hand, the high expression observed for *PDAT1* transcripts in leaf and flower tissues supports the previous findings that *PDAT,* along with *DGAT1,* is essential for normal pollen formation in *Arabidopsis* flowers, and are involved in TAG biosynthesis during seed development [15]. In addition, Camelina *PDAT1* transcripts have similar expression patterns to those of *Arabidopsis* [16] and Castor [17], which support the involvement of *PDAT1* gene in maintaining membrane lipids in seeds and vegetative tissues [17]. Finally, based on the expression profiling of TAG genes examined here, we decided to investigate the complete transcriptome of Camelina seeds at various stages of its development. For this purpose, the seeds harvested at 10–15 DAF, representing early seed stage (termed Cs-14), and at 16–21 DAF, representing the early mid/late seed stage (termed Cs-21) were combined to cover a range of seed development stages for global transcriptome analysis using the RNA-Seq technology.

### Fatty-acid composition and oil accumulation rates during Camelina seed development

**Fig. 2:**
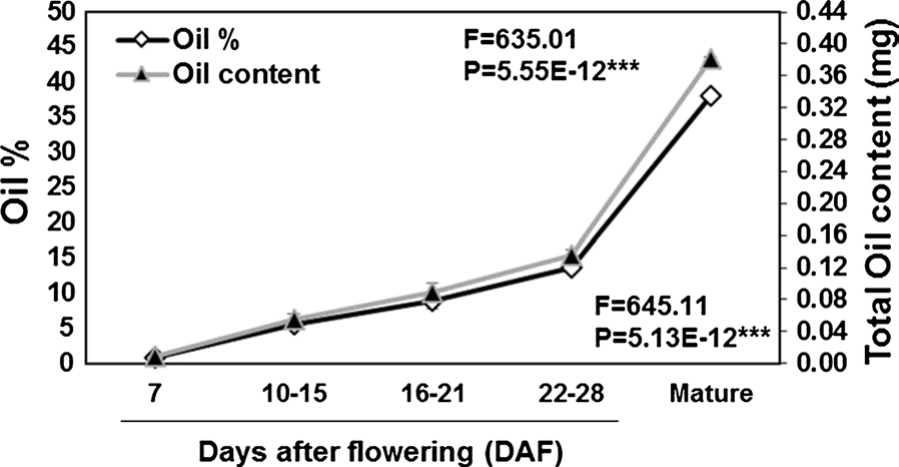
Rate of oil deposition during Camelina seed development. Oil content values are expressed as total oil amount (mg or µg, *gray line*) and  % FW (*black line*). The significance of the effect of developmental seed stages was tested by ANOVA (*F* and *P* values). Data represent the mean of three independent measurements ±SE

To determine whether the expression patterns observed for selected Camelina genes are in agreement with the rate of oil accumulation and the changes in the fatty-acid profile, FAME analysis was applied on Camelina seeds harvested at different development stages. Results revealed significant global and developmental stage-specific changes in the fatty-acid concentrations, their saturation levels, and the amount of oil accumulated (Table 1; Fig. 2, Additional file 1: Fig. S1). Our results indicated that while the oil accumulates in Camelina seeds, the seed water content decreased dramatically throughout seed development. The fatty-acid profile takes its final shape by 28 DAF, with no important changes exhibited beyond this seed stage, although the oil amount continued to increase by almost 65 % in mature seeds, more than its level at this seed stage. At the early seed developmental stage (7 DAF), the seed water content was relatively high (88 % of the total seed weight on average), and the oil content was relatively low (8.6 µg per mg seed on average). Between 10 and 21 DAF, we observed a continuous accumulation of oil, with total oil content accounting for approximately 9 % (w/dry weight), while the seed water content decreasing to about 75 % (w/fresh weight). The seeds harvested at 22–28 DAF showed a significant increase in oil content (~30 %, 285 µg per mg seed), and water contents decreased to 11 %. At the beginning of the seed desiccation phase (from 28 DAF to seeds at maturation), the water content dropped to less than 3 % in mature seeds, whereas oil contents increased massively (~38 %, 380 µg per mg seed). The rapid accumulation of oil starting from 16–21 DAF to seed at maturation (nearly 50 DAF) confirmed the general observation that biosynthesis of storage lipids (i.e., TAGs) usually occur at the mid-late stage of seed development (18–24 DAF) as previously reported [13]. The tremendous increase of oil contents during the course of seed development is consistent with the expression patterns observed for the selected genes examined in this study (Fig. 1). Two genes, *DGAT1* and *LPAT3*, associated with TAG assembly in the Kennedy pathway showed temporal expression patterns, with maximum expression levels at 21 DAF which declined at 28 DAF. This coincides with the stage of seed development where the TAG is rapidly accumulated, suggesting the involvement of these two genes in TAG assembly and accumulation in Camelina developing seeds.

**Table 1:** Fatty-acid composition (%) of Camelina seeds at different stages of development

Fatty acid	Days after flowering (DAF)				Mature seed	Significance *P* value
	7	10–15	16–21	22–28		
C14:0	0.46 ± 0.04	0.00	0.00	0.14 ± 0.03	0.11 ± 0.01	1.78E−07***
C16:0	20.89 ± 0.44	10.40 ± 0.25	9.73 ± 0.20	8.58 ± 0.23	8.04 ± 0.16	4.03E−11***
C16:1	0.39 ± 0.05	0.00	0.00	0.21 ± 0.00	0.16 ± 0.02	5.35E−06***
C18:0	4.19 ± 0.16	5.06 ± 0.15	5.08 ± 0.38	3.79 ± 0.12	3.38 ± 0.08	0.002301***
C18:1	16.02 ± 0.83	17.24 ± 0.9	14.21 ± 0.91	9.95 ± 0.25	11.84 ± 0.15	0.001542***
C18:2	44.01 ± 0.98	29.71 ± 0.11	27.51 ± 0.49	22.24 ± 0.36	21.04 ± 0.14	2.63E−09***
C18:3	8.79 ± 0.42	19.11 ± 0.75	22.17 ± 0.65	29.19 ± 0.62	30.28 ± 0.27	4.39E−10***
C20:0	0.56 ± 0.09	2.16 ± 0.25	3.26 ± 0.14	2.95 ± 0.02	2.44 ± 0.04	4.37E−06***
C20:1	0.77 ± 0.22	11.49 ± 0.33	11.47 ± 0.30	13.64 ± 0.29	12.41 ± 0.19	4.25E−11***
C20:2	0.00	1.57 ± 0.05	1.52 ± 0.15	2.24 ± 0.06	1.96 ± 0.01	1.55E−06***
C20:3	0.00	0.00	0.59 ± 0.03	1.09 ± 0.05	1.11 ± 0.03	0.00023***
C22:0	0.00	0.00	0.67 ± 0.13	0.56 ± 0.06	0.45 ± 0.04	0.000128***
C22:1	0.00	1.68 ± 0.18	2.45 ± 0.19	3.56 ± 0.29	3.45 ± 0.09	2.02E−08***
C24:1	0.00	0.00	0.00	0.61 ± 0.06	0.57 ± 0.01	1.43E−08***
Others	3.90 ± 0.45	1.39 ± 0.18	1.54 ± 0.42	1.21 ± 0.11	2.70 ± 0.04	0.000707***

Data represent the mean ± standard error of three independent measurements. *C14:0* myristic acid; *C16:0* palmitic acid; *C16:1* palmitoleic acid; *C18:0* stearic acid; *C18:1* oleic acid; *C18:2* linoleic acid; *C18:3* α-linolenic acid; *C20:0* arachidic acid; *C20:1* gondoic acid; *C20:2* eicosadienoic acid; *C20:3* eicosatrienoic acid; *C22:0* behenic acid; *C22:1* erucic acid; *C24:1* nervonic acid. The significance of the effect of the developmental stages was tested by ANOVA (*P* values; * *P* < 0.05; ** *P* < 0.01; *** *P* < 0.001)

Furthermore, we studied the changes in the FA composition of total lipids in Camelina developing seeds. As expected, the profile of FA composition during the early stages of seed development differs from that in the mature seeds (Table 1). Early stages showed high levels of palmitic, oleic, and linoleic acid (20.8, 44.01, and 16.02 %), whereas mature seeds contain high levels of α-linolenic and gondoic acid (30.2 and 12.4 %). Among the saturated fatty acids, palmitic, stearic, and arachidic acids were the most abundant in Camelina seeds showing decreased levels of palmitic from 20.8 % in 7 DAF to 8.0 %, arachidic from 0.5 % in 7 DAF to 2.4 % in mature seed, while maintaining a constant level of stearic acid. We also observed major changes in the levels of mono and polyunsaturated fatty acids (named as, MUFA and PUFA, respectively) during seed development. The levels of oleic and linoleic acids were significantly decreased from 16 and 44 % in 7 DAF to 11.8 and 21 %, respectively, in mature seeds. This reduction was associated with the significant increase in α-linolenic acid from 8.7 % in 7 DAF to 30.28 % in mature seed. It is also noteworthy to mention that the levels of very long chain fatty acids (VLCFAs; arachidic, gondoic, eicosadienoic, and erucic) were reached their maximum between 22 and 28 DAF, with gondoic acid (C20:1) represents the most abundant among the VLCFAs. The high accumulation of the α-linolenic acid and the VLCFAs by 28 DAF can be correlated with the stage of seed development where the activities of fatty-acid desaturases (*CsFAD2* and *CsFAD3*) and fatty-acid elongase (*CsFAE1*) enzymes maximized as previously reported by Hutcheon et al. [18],

These observations support the involvement of these two enzymes in desaturation and elongation events occurring in the Camelina developing seeds. In the context of using Camelina oil in human diets, feedstocks, and in biofuels applications, high levels of omega-3 fatty acids (α-linolenic acid) over omega-6 fatty acids (linoleic) are preferable. We detected a 1.5:1 ratio between omega-3 and omega-6, which is close to the recommended ratio (2:1) of using Camelina oil as a nutritional supplement and a general-purpose oil. However, the high level of PUFA in Camelina oil is an undesirable characteristic for most industrial purposes, as they render the oil susceptible to oxidation. This undesirable trait has attracted many researchers to engineer Camelina varieties for improved oil quality containing low PUFA/MUFA ratio [18]. Moreover, the level of erucic acid (C22:1) in Camelina oil was about 3.45 % on an average, and this amount is still below the maximum level (5 %) of erucic acid suggested for human consumption and in foodstuffs containing added oil or fats as well as for biodiesel applications [13]. Therefore, selecting the best Camelina oils is of interest for future industrial applications which would require initiating research programs focusing on engineering Camelina varieties producing oils containing lower ratios of PUFA/MUFA, C20-24/C16-18, and erucic acid (C22:1), and higher long chain PUFA along with increasing seed and oil yields.

### Mapping of RNA-Seq reads against Camelina genome

The Illumina high-throughput next-generation RNA sequencing resulted in millions of short sequencing reads generated from three biological replicates of Cs-14 and Cs-21 samples (Table 2). The mapping results showed that approximately 98 % of the reads were successfully mapped to camelina reference genome and that the majority of reads were mapped in intact pairs (83–93 %), which were further used for gene expression quantification. A small proportion of reads was mapped as broken pairs (11–13 %), and thus excluded from further analysis. Another notable result is that approximately 77 % of the counted pair-end reads were mapped to exonic regions, while the remaining reads were mapped to intronic region (14 %), intergenic regions (7–10 %), and a very small proportion of reads (0.4 %) were defined as unannotated regions, which probably contain novel genes and exons (Table 3). Collectively, the distribution of the mapped reads among different genomic locations showed similar patterns between the two-seed-stage samples; however, the initial sequence read numbers mapped varied. This variation in mapping results can be attributed to the variation in the quality of RNAs and cDNAs used for the analysis as well as the technical variations that may occur during the Illumina sequencing procedures.

**Table 2:** Stages of Camelina seed development and RNA-Seq data sets

	Cs-14 (10–15 DAF)				Cs-21 (16–21 DAF)			
	Bio Rep 1	Bio Rep 2	Bio Rep 3	Mean ± SD	Bio Rep 1	Bio Rep 2	Bio Rep 3	Mean ± SD
Number of sequence reads	53.0 M	54.8 M	78.9 M	62.23 ± 14.46	62.0 M	61.7 M	46.3 M	56.6 ± 8.9
Number of reads after trimming	52.0 M	53.7 M	72.5 M	59.40 ± 11.37	60.7 M	60.5 M	45.4 M	55.5 ± 8.7
Reads mapped in pairs	44.8 M	45.6 M	67.3 M	52.57 ± 12.76	50.7 M	51.1 M	38.6 M	46.8 ± 7.1
Reads mapped in broken pairs	5.9 M	6.7 M	8.4 M	7.00 ± 1.27	8.4 M	7.9 M	5.8 M	7.36 ± 1.3
Reads not mapped	210,281	241,410	372,878	274,856 ± 86,304	319,246	286,780	184,244	263,416 ± 70,466

Shown are the two stages of Camelina seed development used for RNA-Seq. The number of processed reads obtained from three biological replicates at different levels of data analysis of RNA sequencing (in millions M)

*DAF* days after flowering; *SD* standard deviation

**Table 3:** Distribution of sequencing reads among different genomic locations

	Cs-14 (10–15 DAF)				Cs-21 (16–21 DAF)			
	Bio Rep 1	Bio Rep 2	Bio Rep 3	Mean ± SD	Bio Rep 1	Bio Rep 2	Bio Rep 3	Mean ± SD
Annotated exons	17.2 M (76 %)	17.5 M (76 %)	25.5 M (75 %)	20.0 ± 4.7	19.4 M (77 %)	19.2 M (75 %)	14.9 M (77 %)	17.8 ± 2.5
Annotated introns	3.3 M (14 %)	3.4 M (15 %)	4.8 M (14 %)	3.8 ± 0.8	3.6 M (14 %)	3.5 M (14 %)	2.7 M (14 %)	3.2 ± 0.4
Novel genes and exons	21,028 (0.4 %)	24,141 (0.4 %)	37,287 (0.5 %)	27,485 ± 8630	31,924 (0.5 %)	28,678 (0.4 %)	18,424 (0.4 %)	26,342 ± 7046
Intergenic	1.8 M (8 %)	1.8 M (7 %)	3.3 M (10 %)	2.3 ± 0.8	2.3 M (9 %)	2.7 M (10 %)	1.6 M (8 %)	2.2 ± 0.5
Total unique reads	22.4 M	22.8 M	33.6 M	26.2 ± 6.3	25.3 M	25.5 M	19.3 M	23.3 ± 3.5

Shown are the number and percentage of sequencing read mapping to the indicated genomic regions. Novel gene and exon regions refer to the reads mapped to previously unannotated regions that are enriched in sequencing reads. The number of processed reads obtained from the mapping of the three biological replicates of RNA sequencing to different regions of Camelina genome (in millions M)

*DAF* days after flowering; *SD* standard deviation

### RPKMs assignment and transcript abundance

**Fig. 3:**
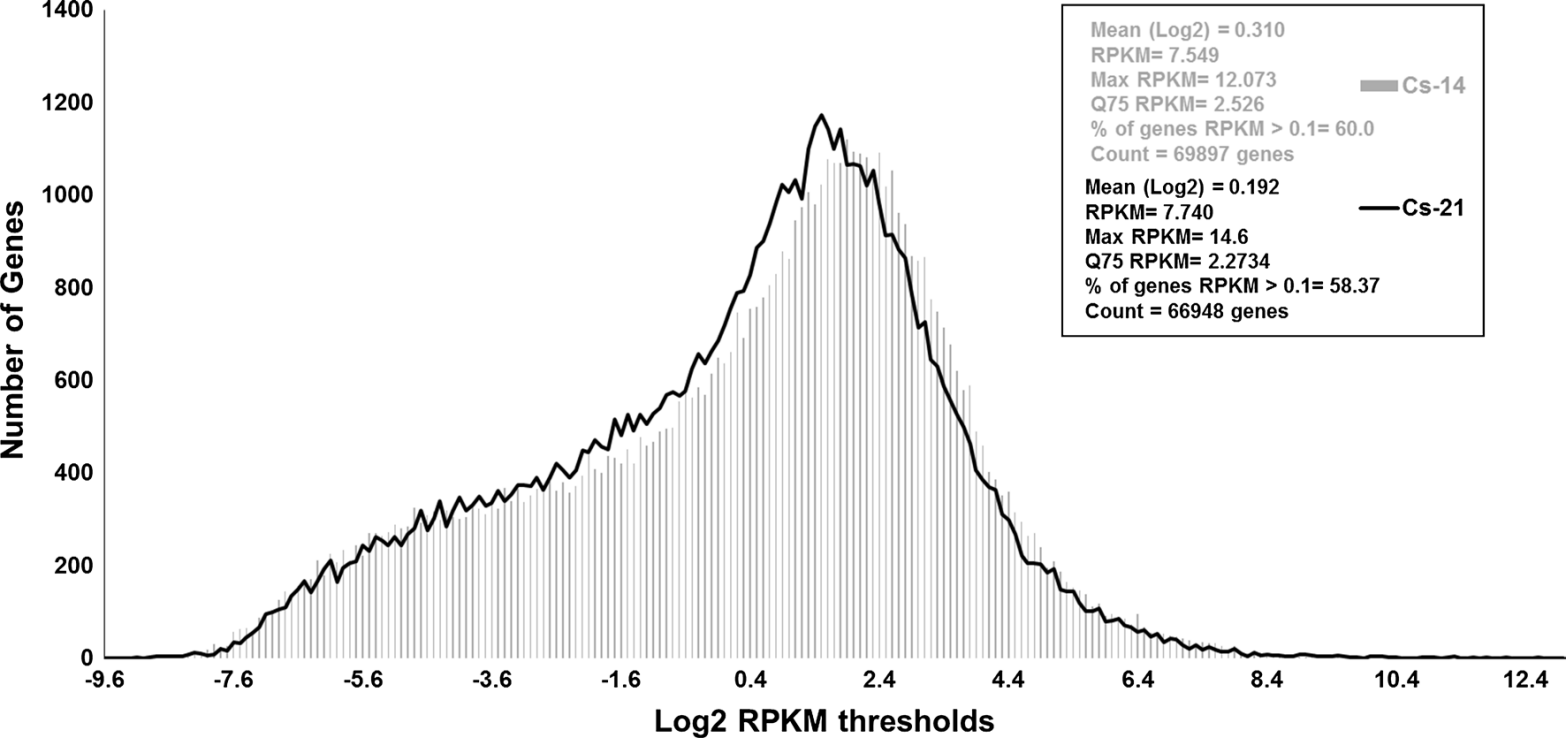
Distribution of RPKM values (in log2 scale) for the genes identified in Camelina developing seeds. The means for both the original and log2 transformed RPKMs, maximum RPKMs, 75 % percentile (Q75), the total number of genes analyzed, and the genes with RPKM >0.1 are shown

To detect the number of actively expressed genes in the Camelina transcriptome, the normalized RPKMs (in log2 scale) were used as expression values, and the number of transcripts expressed at each RPKM threshold was estimated (Fig. 3). The density plot shown in Fig. 3 indicates that Camelina transcripts were expressed in similar patterns in early (Cs-14) and late (Cs-21) seed stages, despite the initial variation observed in the number of transcripts tested (69,897 and 66,984 transcripts in Cs-14 and Cs-21, respectively).

Furthermore, application of a threshold value of RPKM = 0.1 resulted in approximately 42,004 (60 %) and 39,084 (58.3 %) transcripts being actively expressed (RPKM ≥ 0.1) in both Cs-14 and Cs-21, respectively (Fig. 3). Among the actively expressed genes detected, approximately 156 genes in Cs-14 and 188 genes in Cs-21 were expressed in high abundance (RPKM > 7.75) when normalized to the expression level of a housekeeping gene, ubiquitin-conjugating enzyme 10 (*UCE10*). Approximately 4038 and 6272 genes were highly expressed (RPKM > 4.47) in Cs-14 and Cs-21, respectively, when normalized to the expression of the housekeeping gene β-*actin*. These highly expressed genes were shown to encode proteins belonging to various protein families, including protease inhibitors, seed storage proteins, lipid transfer proteins, and plant defense-associated proteins (see Additional file 2: Table S2, Additional file 3: Table S3).

The observation that only 58–60 % of the genes, which were previously identified in the Camelina reference genome (total of 89,418 genes), could be detected in our transcriptome analysis (RPKM > 0.1) may contribute to the tissue-specific sampling approaches followed in the present study. Our transcriptome covers the genes that are exclusively expressed in developing seeds between 10 to 21 DAF, and we expected a lower number of genes to be detected. This is because the Camelina genome, used here as a guide in mapping and annotation, is widely covered for the transcripts obtained from RNA pools of 12 different Camelina tissues [19]. Furthermore, as previously reported, the polyploidy nature of Camelina genome may also influence the expression levels of the genes with more than two copies, leading to down-regulation and/or silencing of some duplicated genes either throughout the plant or in a tissue-specific manner [18].

Furthermore, to examine whether Camelina transcriptome is exhibiting tissue-specific gene expression pattern, we compared the expression levels of all detected transcripts among seed and leaf tissues. Figure 3 shows the number of expressed genes detected in Camelina seeds and leaf tissues (RPKM ≥ 0.1). According to the figure, the majority of Camelina transcripts (85 %) were found to be ubiquitously expressed in the seed and leaf tissues, but with differential and temporal expression patterns between the tissues. These transcripts probably contribute to the key traits required to maintain the phenotype and tissue profile in Camelina.

**Fig. 4:**
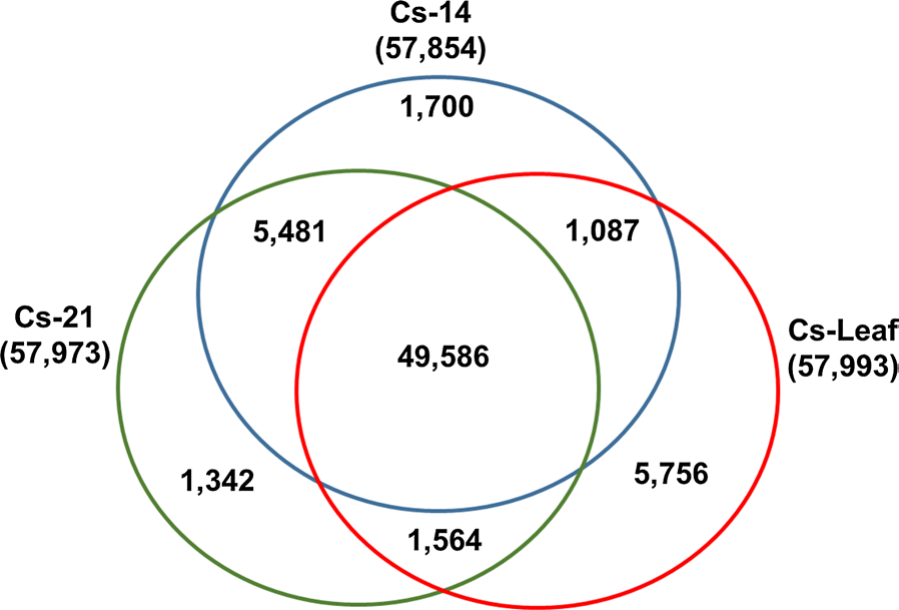
*Venn diagram* showing the actively expressing genes in developing seeds and leaf tissues. Among the genes, 49,586 are expressed in seeds and leaf tissues, 5481 are co-expressed in Cs-14 and Cs-21, 1087 are co-expressed in Cs-14 and leaf, and 1564 are co-expressed in Cs-21 and leaf. The number of tissue—specifically expressed genes—is 1700 (Cs-14), 1342 (Cs-21), and 5756 (leaf), respectively. Cs-14: 10–15 DAF; Cs-21: 16–21 DAF; Cs-Leaf: leaf

It was also noticed that approximately 8523 genes showed seed-specific expression in comparison to 5756 genes found to be expressed exclusively in leaves (Fig. 4). Among the seed-specific genes tested, we found the majority encode proteins involved in lipid and reserve storage, including oleosins, caleosins, cruciferins, and 2S albumins, while a high proportion of leaf-specific genes encode ribulose-1,5-bisphosphate carboxylase/oxygenase (RuBisCO) protein family (see Additional file 3: Table S3; Cs-14, Cs-21, and Cs-Leaf sheets).

### Estimation of the differentially expressed genes (DEGs)

**Fig. 5:**
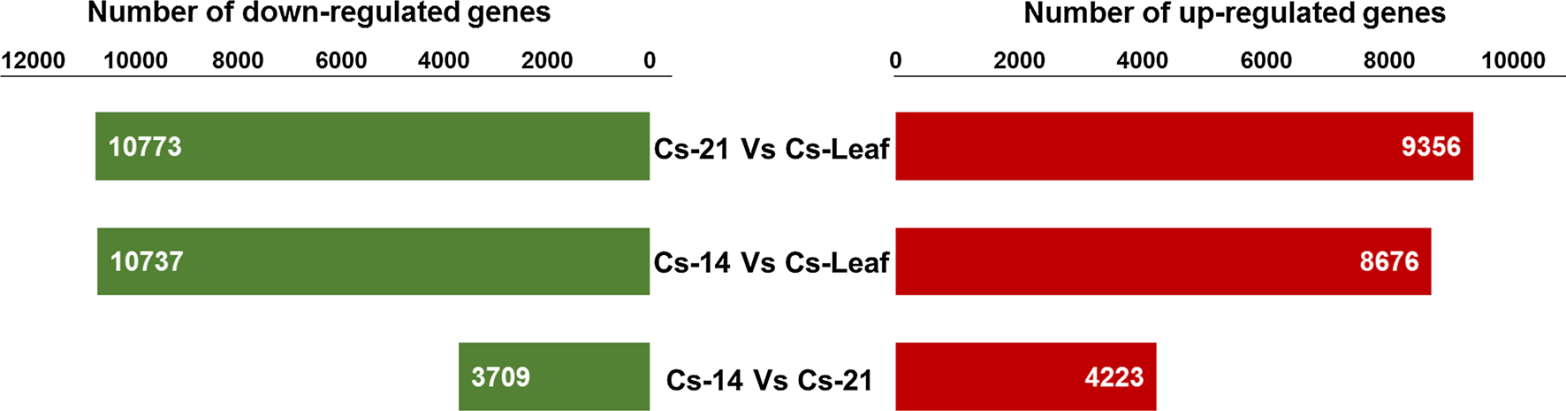
Changes in gene expression profiles among the different developmental stages of Camelina seeds and leaf tissues. The number of differentially expressed genes between Cs-14 and Cs-21, Cs-14 and Cs-Leaf, Cs-21 and Cs-Leaf, is summarized. Between Cs-14 (10–15 DAF) and Cs-21 (16–21 DAF), there are 4223 genes up-regulated and 3709 genes down-regulated, between Cs-14 and Cs-Leaf, there are 8676 genes up-regulated and 10,737 genes down-regulated, while there are 9356 genes up-regulated and 10,773 genes down-regulated between Cs-21 and Cs-Leaf. Cs-14 sample was used as a control in Cs-14 vs Cs-21 comparison, while Cs-Leaf sample was used as a control in both Cs-14 vs Cs-Leaf and Cs-21 vs Cs-Leaf comparisons

To identify the Camelina genes that are differentially expressed during the seed development and between different Camelina tissues, we compared and then statistically analyzed the expression levels of Camelina transcripts (Log2 RPKMs) in developing seeds and in leaf tissues. The volcano plots (Additional file 1: Fig. S2) distribute the RPKM values for the transcripts among different fold changes (in log2 scale, *X*-axis), and different significance levels (*P* value, *Y* axis). On this basis, we selected the transcripts with fold changes ≥1.5 or ≤−1.5 and *P* values ≤0.05 as DEGs. This method enabled us to identify approximately 7932 DEGs, which significantly changed during the seed development and 39,542 DEGs, which dramatically changed when seed and leaf data were compared (Fig. 5). During the seed development, using Cs-14 data as a control, the expression levels of the 4223 transcripts were significantly increased as the seeds develop in Cs-21 (16–21 DAF) compared to 3709 transcripts up-regulated at earlier seed stage in Cs-14 (10–15 DAF). These findings can be attributed to the demand for metabolic changes at later stages of seed development where lipids and other storage compounds deposit in the seeds. Moreover, a comparison between seed and leaf data, using leaf data as a control, revealed a total of 8676 (in Cs-14) and 9356 (in Cs-21) transcripts, which showed a significant increase in their expression levels as compared to their levels in the leaf tissue. Full lists of the DEGs with their descriptions and expression levels are summarized in (see Additional file 4: Table S4; RPKM, Cs-14 vs Cs-21, Cs-14 vs Cs-Leaf, and Cs-21 vs Cs-leaf sheets).

### Annotation of DEGs and functional categories determination

**Fig. 6:**
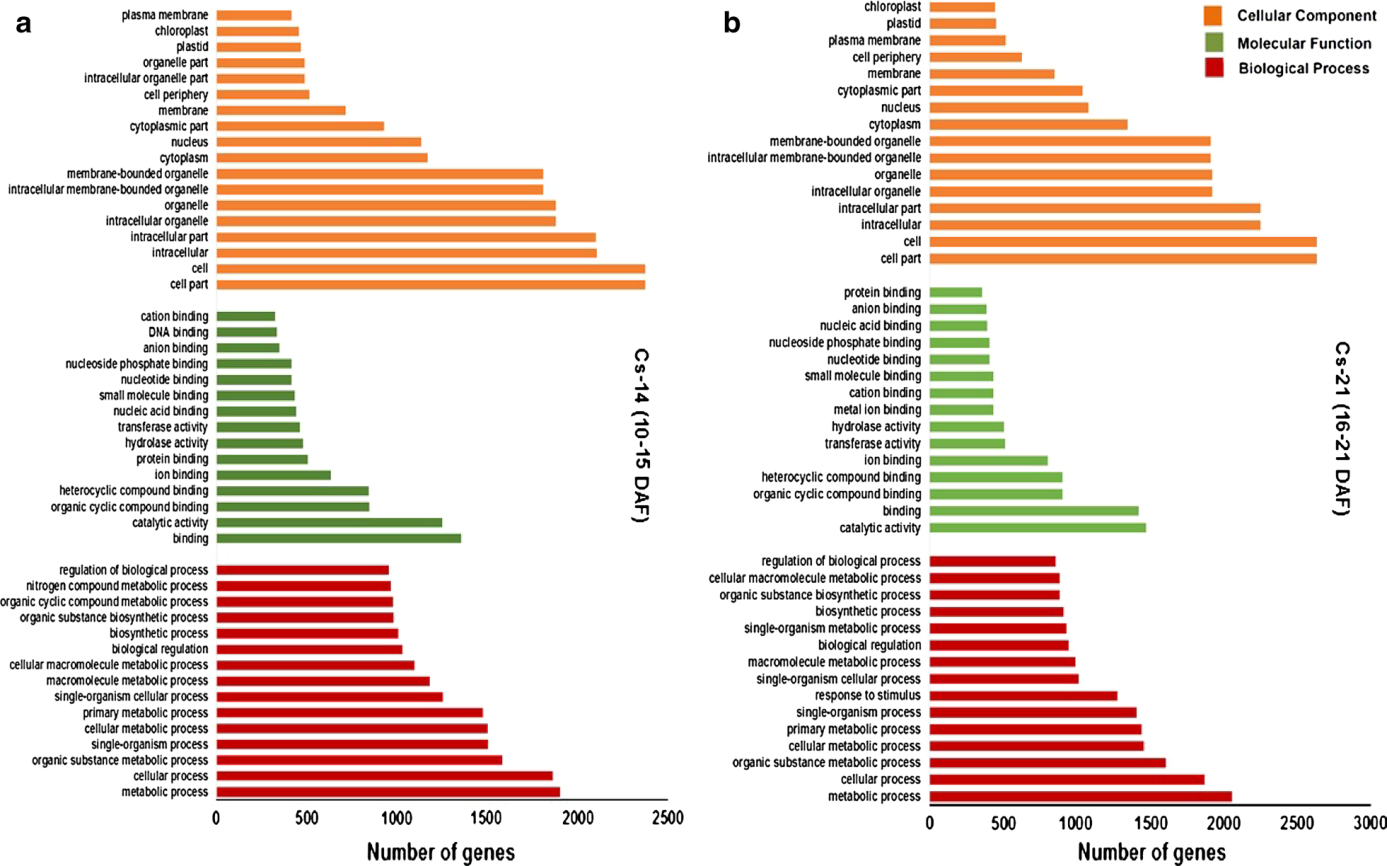
GO classification: Gene ontology distribution of the differentially expressed transcripts in Camelina developing seeds. Cs-14 (**a**) and Cs-21 (**b**). The results are summarized in three main categories of GO classification; biological process, molecular function, and cellular component

To determine the global patterns of gene expression and highlight the involvement of the identified DEGs in lipid metabolism during Camelina seed development, the DEGs were annotated and were categorized into various GOs. The GO classification results indicated that the DEGs are involved in different biological processes and molecular functions in distinct cell compartments (Fig. 6). We found that the majority of transcripts encoded proteins regulating various metabolic/biosynthetic processes, including those involved in lipid metabolism. Among these transcripts, many showed binding activity for ions, proteins, and nucleic acids. Furthermore, many transcripts were found to encode hydrolase and transferase enzymes. A total of 985 and 972 transcripts were identified as hydrolases and transferases, respectively. We highlighted these two enzyme groups, as they may play critical roles in determining the rate of lipids degradation and lipids synthesis, respectively, in Camelina seeds. The functional categories for the identified DEGs are summarized in Additional file 6: Table S6 and Additional file 7: Table S7.

In context to the importance of understanding the molecular basis of oil synthesis in Camelina seeds, we examined the changes in expression abundance for genes-encoding proteins involved in acyl lipid metabolism, with emphasis on TAG synthesis and degradation. Overall, GO annotation of the DEGs having lipid metabolism-related functions resulted in relatively 1281 and 1239 genes expressed (RPKM > 0.1) in Cs-14 and Cs-21, respectively, which were annotated as key players in various levels of lipid metabolism, particularly in fatty acids, glycerolipids, and glycerophospholipids biosynthesis (Additional file 5: Table S5). Among those, approximately 50 genes were involved in glycerolipid metabolism, while 34 genes were involved in glycerophospholipid metabolism in both Cs-14 and Cs-21 (see Additional file 6: Table S6, Additional file 7: Table S7, KEGG pathways sheets).

Furthermore, to emphasize the critical factors in oil synthesis, accumulation, and degradation in Camelina, we specifically highlighted the transcripts with molecular functions related to TAG synthesis and degradation (Table 4). As shown in the table, the majority of the transcripts were annotated as acyltransferases (51 genes in Cs-14 and 76 genes in Cs-21), with a developmental increase in the number of acylglycerols, glycerol-3-phosphate, and diacylglycerol acyltransferases during the seed development (54 transcripts in Cs-21 compared to ten transcripts in Cs-14). On the other hand, the number of transcripts with lipase activities doubled, as the seeds develop (35 genes in Cs-21 compared to 17 genes in Cs-14), including those targeting TAGs. This indicates radical changes in Camelina transcriptome associated with synthesis and accumulation of lipid components into the seeds, particularly at later developmental stages, reflecting the temporal and developmental expressions of TAG-related genes. During early embryogenesis, fatty-acid components undergo synthesis and degradation cycles to provide carbon and other nutrients used mainly for rapid cell division and embryo growth [20]. This might be a reason for the increasing number of hydrolases and lipases acting on ester bonds, providing free fatty acids and glycerol via de-esterification. As Camelina seed develops, the resources are allocated to synthesize storage compounds, including TAG and its components. As a result, the committed acylation steps, which utilize glycerol-3-phosphate (G3P) and acyl-CoA pools to ultimately produce TAGs, require the activity of acyltransferases. Altogether, these findings suggest that there are considerable differences between the physiological processes in the early and middle stages of Camelina seed development.

**Table 4:** GO classification for the differentially expressed genes, DEGs, involved in lipid biosynthesis-related functional categories during Camelina seed development with emphasis on the transcripts playing roles in fatty-acid and triacylglycerol biosynthesis/degradation

GO classification (terms)	Term (accession)	Number of transcripts	
		Cs-14 (10–15 DAF)	Cs-21 (16–21 DAF)
Transferase activity, transferring acyl groups	GO:0016746	51	76
2-acylglycerol O-acyltransferase activity	GO:0003846	–	1
Acylglycerol O-acyltransferase activity	GO:0016411	1	9
Diacylglycerol O-acyltransferase activity	GO:0004144	1	8
Glycerol-3-phosphate O-acyltransferase activity	GO:0004366	2	–
Glycerol-3-phosphate 2-O-acyltransferase activity	GO:0090447	–	12
Long-chain-alcohol O-fatty-acyltransferase activity	GO:0047196	–	1
O-acyltransferase activity	GO:0008374	6	23
Lipase activity	GO:0016298	17	35
Acyl-CoA hydrolase activity	GO:0047617	–	2
Acylglycerol lipase activity	GO:0047372	–	1
Fatty-acid ligase activity	GO:0015645	2	5
Long-chain fatty-acid ligase activity	GO:0008922	2	5
Phospholipase activity	GO:0004620	4	15
Phospholipase C activity	GO:0004629	2	5
Phospholipase D activity	GO:0004630	1	4
Triglyceride lipase activity	GO:0004806	10	11
Very long-chain fatty-acid-CoA ligase activity	GO:0031957	–	3
Lipid binding	GO:0008289	42	43
Phospholipid binding	GO:0005543	15	6
Diacylglycerol kinase activity	GO:0004143	3	1
Fatty-acid elongase activity	GO:0009922	2	4
Fatty-acid synthase activity	GO:0004312	2	5
Fatty-acid transporter activity	GO:0015245	2	1
Fatty-acyl-CoA reductase (alcohol-forming) activity	GO:0080019	1	4
Long-chain-fatty-acyl-CoA reductase activity	GO:0050062	1	4
Phosphatidylcholine 1-acylhydrolase activity	GO:0008970	1	2
Lipid transporter activity	GO:0005319	9	4
Phospholipid transporter activity	GO:0005548	6	1

The Gene ontologies GO classification of the selected protein families involved in lipid biosynthesis is shown with the number of transcripts belongs to each functional category during Camelina seed development is presented. Cs-14 represents the early stage of seed development at 10–15 DAF days after flowering, while Cs-21 represents the late stages of seed development at 16–21 DAF

### Expression profiling for gene networks involved in fatty acid and TAG biosynthesis

The comparative transcriptome approach using normalized RPKMs as expression values was applied to RNA-Seq data from Camelina seeds and leaf tissues and the candidate transcripts involved in fatty-acid (FA) synthesis in plastids and TAG assembly in the endoplasmic reticulum ER were highlighted. Additional file 1: Table S8 showed the dominant DEGs significantly changed during the seed development (Cs-14 vs Cs-21), and between developing seeds and leaf tissue (Cs-14 vs Cs-Leaf and Cs-21 vs Cs-Leaf). According to the table, the transcripts-encoding seed storage protease inhibitors and lipid transferases are significantly up-regulated in Cs-14, while transcripts activated in response to abiotic and oxidative stresses as well as dehydration damage are significantly up-regulated in Cs-21. Between seeds and leaf tissues, the expression of the transcripts involved in homeostasis of calcium, seed germination, flowering, gene maturation, and oleosins has significantly increased in developing seeds compared to leaf, whereas the transcripts associated with carbon fixation pathways (i.e., RuBisCO) are dominant in leaf tissues (Additional file 1: Table S8).

**Fig. 7:**
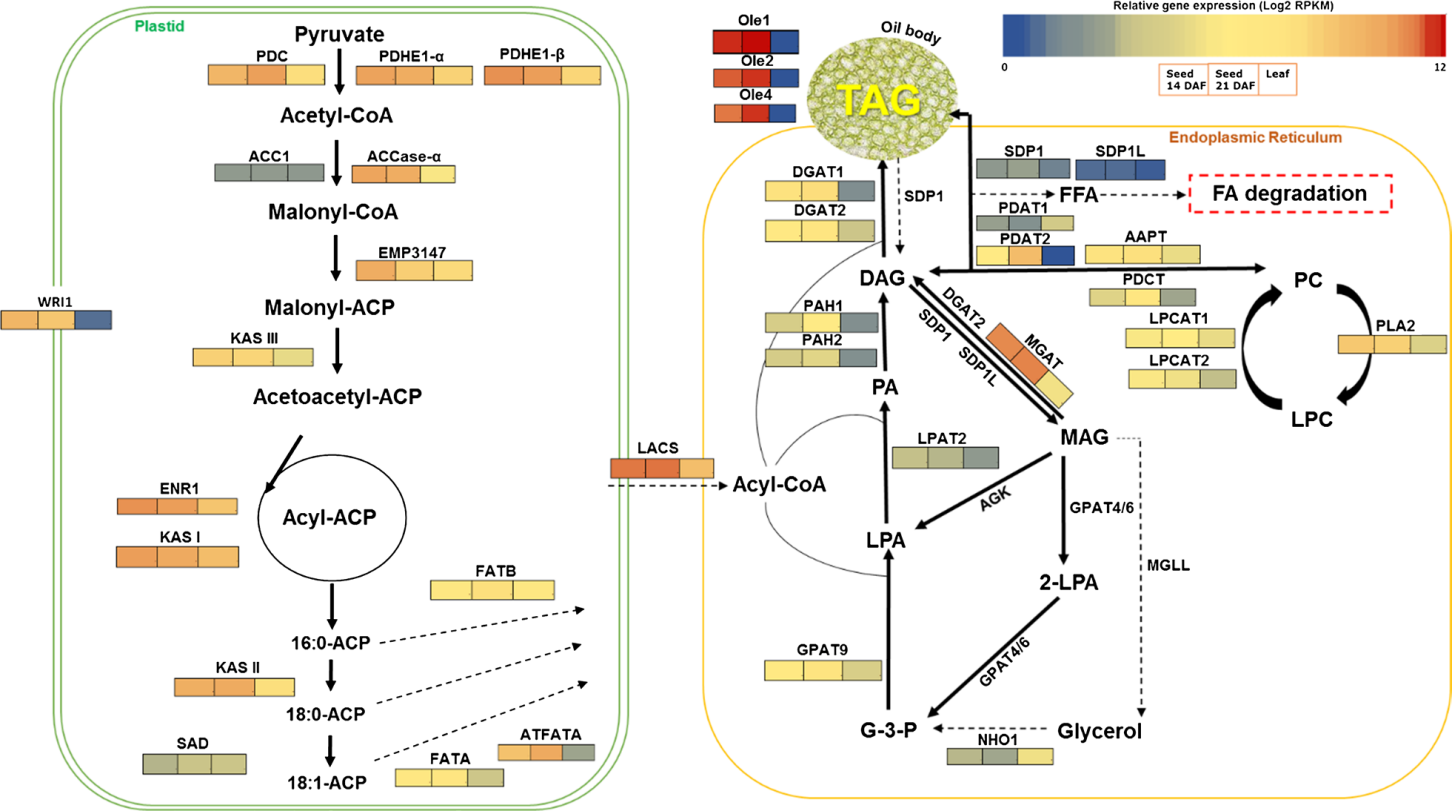
Working model for the genes/gene networks involved in fatty acid and TAG biosynthesis in *Camelina sativa*. The expression abundance (RPKMs in log2 scale) for the selected candidate genes are highlighted in different color scales in Camelina developing seeds at 10–15 days after flowering (Seed 14 DAF) and at 16–21 DAF (seed 21 DAF) as well as in leaf tissues. The full name for the metabolites shown in the pathways is *pyruvate*, *acetyl*-*CoA*, *malonyl*-*CoA*, *malonyl*-*ACP* acyl-carrier protein, *Acetoacetyl*-*ACP*, *Acyl*-*ACP*, sn-Glycerol 3-phosphate G3P, *LPA* lysophosphatidic acid, PA Phosphatidic acid, *MAG* monoacylglycerol, *FAA* free fatty acid, *DAG* 1,2-Diacylglycerol, *TAG* Triacylglycerol, *PC* phosphatidylcholine, *LPC* lysophosphatidylcholine, and *glycerol*. The enzymes shown here are pyruvate dehydrogenase E1-α (*PDH*-*E1*-*α*), *PDH*-*E1*-*β* pyrxuvate dehydrogenase E1-β, *PDC* pyruvate dehydrogenase complex, *ACC1* Acetyl-CoA carboxylase, *ACCase*-*α* acetyl-CoA carboxylase, a-carboxyltransferase, *EMP3147* acyl-carrier-protein S-malonyltransferase, *KASI* ketoacyl-ACP synthase I; *KASII* ketoacyl-ACP Synthase II; *KASIII* ketoacyl-ACP synthase III, *WRl1* wrinkled 1, *ENR1* enoyl-ACP reductase, *ATFATA* fatA acyl-ACP thioesterase, *FATA* acyl-ACP thioesterase A, *FATB* fatty-acyl-ACP thioesterase B, *SAD* stearoyl-ACP desaturase, *LACS* long chain Acyl-CoA synthase, *NHO1* protein-similar to glycerol kinase, *MGAT* monoacylglycerol acyltransferase, *AAPT1* Choline/ethanolaminephosphotransferase, *GPAT9* glycerol-3-phosphate acyltransferase 9, *LPAT2* lysophosphatidyl acyltransferase 2, *SDP1* sugar-dependent protein, *SDP1*-*L* sugar-dependent 1-like protein, *PAH1* phosphatidic acid phosphohydrolase 1, *PAH2* phosphatidic acid phosphohydrolase 1, *DGAT1* diacylglycerol O-acyltransferase 1, *DGAT2* diacylglycerol O-acyltransferase 2, *PDAT1* phospholipid:diacylglycerol acyltransferase 1, *PDAT2* phospholipid:diacylglycerol acyltransferase 2, *DGK* diacylglycerol kinase, *AGK* acylglycerol kinase, *MGLL* acylglycerol lipase, *PDCT* phosphatidylcholine: diacylglycerol cholinephosphotransferase, *AAPT* aminoalcoholphosphotransferase, *LPCAT1* lysophosphatidylcholine acyltransferase 1, *LPCAT2* lysophosphatidylcholine acyltransferase 2, *PLA2* phospholipase A2, *Ole1* Oleosin1, *Ole2* Oleosin 2, and *Ole4* Oleosin 4. This model is modified from the model published by Wang and colleagues in 2012 [44], Dussert and colleagues in 2013 [45], and the glycerolipids metabolism pathway in KEGG database http://www.genome.jp/kegg/. The Gene/enzyme names are modified from the names available in the TAIR database (http://www.Arabidopsis.org) and the Camelina genome database (http://www.camelinadb.ca)

Furthermore, with the emphasis on FA and TAG biosynthesis, the expression abundance of 53-related genes was estimated, and the impact of these genes on alternate metabolic routes for TAG synthesis and accumulation was illustrated (Fig. 7; Additional file 1: Table S9). The genes are grouped herein by their putative functions, independent from their relative expression levels.

#### Fatty-acid synthesis in plastids

Overall, the results indicate high and developmentally regulated levels of transcription observed for the candidate genes involved in FA synthesis, particularly at early seed stages (10–15DAF). These genes participate in acetyl-CoA formation (i.e., pyruvate dehydrogenase complex (*PDC*), the two subunits of Pyruvate dehydrogenase (*PDHE1*-*α* and *PDHE1*-*β*), fatty-acid synthesis (i.e., Acetyl-CoA carboxylase (*ACC1*), Acetyl-CoA carboxylase (*ACCase*-*α*), ketoacyl-ACP synthase (*KASs*)), acyl chain termination and release (i.e., Acyl-ACP thioesterase A and B—*FATA* and *FATB*), and activation of long-chain fatty acids (i.e., long-chain acyl-CoA synthase—*LACS*). Focusing on the enzymes involved in *de novo* formation of acyl chains in plastids, the two enzyme systems namely acetyl-CoA carboxylase (*ACCase*) and fatty-acid synthase (*FAS*), were highlighted. In this regard, the genes encode the alpha subunit of the carboxyltransferase named as *ACCase*-*α*, which converts acetyl-CoA into malonyl-CoA, and the three fatty-acid synthases namely, 3-ketoacyl-ACPs (*KASI*, *KASII*, and *KASIII*), which are responsible for the condensation of acetyl-CoA and malonyl-ACP, showed ubiquitous expression, but with higher levels of expression in seeds compared to leaf (Fig. 7, Additional file 1: Table S9). Furthermore, since the transfer of the acyl chain from the ACP via hydrolysis terminates the reactions of the fatty-acid synthesis, we highlighted the expression of acyl-ACP thioesterases, which catalyze this step. The two *Arabidopsis* homologs *FatA* (At3g25110 and At4g13050) and *FatB* (At1g08510) genes showed extensive expression abundance, especially during the seed development. The transcription of *FatA* gene was significantly elevated in late seed stages (Cs-21), and this increase of *FatA* gene expression, which involves in C18 fatty-acid synthesis, compared to *FatB*, which releases C16 fatty acids, may reflect demand for C18 fatty acids for seed oil synthesis [21].

Finally, to emphasize the key regulators of fatty-acid synthesis in plastids, the transcriptional activity of the transcription factors *WRl1*, which has been shown to regulate the activity of many FA synthesis genes, was highlighted. *WRl1* transcripts showed high and exclusive activity in developing seeds, especially at early seed stages (Additional file 1: Table S9). The expression pattern observed for *WRl1* transcripts can be contributed to the increased levels of the major genes for FA synthesis. For example, the two FA synthesis-related genes *ACCase*-*α* and enoyl-ACP reductase (*ENR1*) exhibit altered expression levels in response to the up- or down-regulation of *WRl1* expression. This observation supports the previous findings that these two genes appeared to be putative targets of *WRl1* [22] and that any change in the regulation of *WRl1* would probably lead to significant increase in fatty-acid supply from plastids and ultimately the amount of TAG produced. To confirm this, the *ArabidopsisWRl1* gene was overexpressed in Camelina under seed-specific promoters and the transgenic lines produced higher seed and oil yields compared to controls [23], thus providing a new candidate to improve Camelina oil and seed qualities. Therefore, the high activity of the genes associated with FA biosynthesis would lead to the availability of fatty-acyl-CoA pools that are exported from the plastid to be further utilized for glycerolipid metabolism, including TAGs, in the ER.

#### Triacylglycerol synthesis

While the ‘push’ activity of *WRl1* provides the pool of acyl CoAs into the cytoplasm, a ‘pull’ activity by the enzymes in the ER membranes should be integrated to initiate TAG assembly via the Kennedy Pathway, while limiting the flux of acyl chains into competitive metabolic pathways. To examine this, we highlighted the expression profile of candidate genes attributed to TAG assembly in the ER and grouped them into three categories; synthesis of membrane lipids in ER, oil synthesis, and oil storage. Overall, there is a significant increase in the expression levels of acyltransferases, which play critical roles in TAG synthesis utilizing various metabolic routes, including *GPAT9*, *DGAT1*, *DGAT2*, *PDAT2*, *LPCAT1*, *LPCAT2*, and *MGAT*, with maximum relative expression (MRE) equals to 1.14, 1.47, 1.01, 2.14, 0.76, 0.75, and 10.34, respectively, relative to the expression of *β*-*actin* (see Additional file 1: Table S11). This high activity for acyltransferases seems to cause an impact on the amount of TAG and glycerolipids accumulated as well as their fatty-acid composition, as these enzymes act as sinks for the supply for acyl-CoA pools and glycerol exported from plastids.

In Camelina, like in other oilseed crops, the TAG assembly appears to be initiated by the activity of GPATs to esterify the acyl group from acyl-ACP to the *sn*-1 of glycerol-3-phosphate producing LPA, and LPATs to convert the resulted LPA to PA. The latter is dephosphorylated to form DAG by the activity of phosphatidic acid phosphatases (*PAPs*). Accordingly, the expression of the *Arabidopsis* homologs of *GPATs* and *LPATs* was detected and quantified (Table S9). Among the nine GPATs identified in Camelina (termed GPAT1-9), only the ER-localized *GPAT9* transcripts were shown to be expressed at high levels, particularly in developing seeds. Their expression patterns provide further evidence for their probable roles in lipid storage as previously reported [24, 25]. Furthermore, the expression abundances of the five *Arabidopsis* homologs of LPATs (termed LPAT1-5) were also measured in Camelina. Surprisingly, the ER-localized *LPAT2*, which is previously reported to be involved in TAG synthesis in *Arabidopsis* and Brassica [26], showed lower expression levels in developing Camelina seeds, with the exception of one genomic copy (Csa04g044150, see Table S9). Another LPAT (termed *LPAT1*) that encodes the plastid LPAT showed ubiquitous expression, with high levels in leaf tissue (Table S9). This LPAT was reported to be essential for embryo development, and when its function disrupted, it led to embryo death [27]. The expression patterns observed for Camelina LPATs seem not to support their activities in maintaining the metabolic flow of LPA into different PAs, and in determining the amount of DAGs accumulated in Camelina seeds. Therefore, further studies are needed to characterized the role of LPATs in TAG biosynthesis in Camelina to determine which LPAT isoforms are directly involved in TAG assembly and accumulation into the ER.

Since DAGs are the intermediate precursor molecules to TAG synthesis, the model presented in Fig. 6 highlighted different metabolic routes for DAG synthesis in Camelina. The de novo DAG synthesis seems to be initiated by the dephosphorylation of PA to produce DAG by the activity of different *PAPs*. In this context, the two ER-localized *Arabidopsis* homologs of PAPs, *PAH1* (AT3G09560), and *PAH2* (AT5G42870) showed relatively high expression levels in developing seeds (MRE equal to 0.9 and 0.6, respectively, relative to *β*-*actin*, Additional file 1: Table S9). The expression patterns observed for *PAH1* and *PAH2* appear to be consistent with their roles in lipid synthesis in *Arabidopsis* and rapeseed [28], in addition to their activities in regulating membrane phospholipids [29, 30].

DAG can also be de novo synthesized from the acylation of MAG via the activity of the bifunctional enzyme *MGAT*, which showed high expression abundance in developing seeds (MRE equal to 10.43 relative to *β*-*actin*, Additional file 1: Table S11). This expression pattern for *MGAT* transcripts agrees with the possibility of *MGAT* activities in MAG-to-DAG conversion, which was initially hypothesized by Vijayaraj et al. [31]. Beside the acyltransferase activity of *MGAT*, it was also shown that *ArabidopsisMGAT1* acts as a lipase, which could hydrolyze MAG and lysophosphatidylcholine (LPC) as substrates [32]. This may require MGAT enzyme to be actively expressed at an early seed stage (i.e., 14 DAF) to provide the substrates for *DGAT1*, which, in turn, become abundant at later seed stage (i.e., 21 DAF, see Additional file 1: Fig. S1). However, further studies are needed to explain the potential roles of *MGATs* in TAG biosynthesis into Camelina seeds.

In an alternate metabolic route, DAG is synthesized from the conversion of the membrane lipid phosphatidylcholine (PC) into DAG. This interconversion has been shown to be catalyzed by the activities of the two enzymes: aminoalcoholphosphotransferase (*AAPT*) and phosphatidylcholine: diacylglycerol cholinephosphotransferase (*PDCT*). The transcripts encoding *AAPT* and *PDCT* enzymes also showed high expression levels (MRE equals to 0.96 and 0.93, respectively, relative to β-actin, Additional file 1: Table S11). These expression patterns detected for *PDCT* and *AAPT* genes indicate their potential activities to provide DAG pools enriched with PUFA, which are later incorporated into the accumulated TAGs via the activities of *DGATs* or *PDATs*. Therefore, any changes in *PDCT* and/or *AAPT* enzyme activities probably affect directly or indirectly both the levels of FA saturation and the quantities of TAGs produced.

Furthermore, the rate-limiting step in converting DAG to TAG seems to be catalyzed by the activities of *DGATs* and/or *PDATs*. Camelina transcripts quantification indicated the active expression of four *Arabidopsis* homologs of DAG acyltransferases, namely *DGAT1*, *DGAT2*, and *PDAT1* and its closest homolog *PDAT2* (At3g44830, see Additional file 1: Table S9). Both *DGAT1* and *DGAT2* showed ubiquitous and high expression levels, particularly in developing seeds (MRE equals 1.47 and 1.0, respectively, relative to β-actin), with consistent expression patterns for both *DGAT1* and *DGAT2* from Cs-14 through Cs-21. Furthermore, we detected expression of *PDAT1* at higher levels in leaf than its levels in developing seeds. *PDAT2* was exclusively expressed in developing seeds, in high levels (MRE equals to 2.14 relative to β-actin), while no transcript was detected in leaves. The prolonged expression patterns detected for *DGAT1*, *DGAT2*, and *PDAT2*, during the seed development, can be contributed to their enzyme kinetics that probably depend upon the demands of DAG-to-TAG conversion, although little is known about the contribution of *DGAT2* and *PDAT2* for oil synthesis in oilseed crops. However, previous studies suggested that *DGAT1* seems to be the rate-limiting enzyme in TAG metabolism in different plant species, and its increased levels have led to significant increase in oil contents, seed yield, and germination rates in transgenic lines [33–35]. Therefore, the increased expression levels *DGAT2* and *PDAT2* transcripts in Camelina developing seeds should not be considered strong evidence for the involvement of those genes in TAG biosynthesis unless further research is conducted to characterize their functions in TAG synthesis. Finally, the low expression levels detected for *PDAT1* in Camelina seeds is not consistent with the previous findings that *PDAT1* enzyme can catalyze TAG formation, in the absence or along with *DGAT1*, under any circumstances, and that it appears to be essential for normal development of both seeds and pollen [3].

#### Oleosins and seed storage proteins

Many transcripts were identified and annotated as oil body proteins known as oleosins, which contribute to oil body biogenesis and stabilizing TAG/cytosol oil body interface [36]. Here, we highlighted the expression patterns for the three *Arabidopsis* oleosins homologs named *Ole1*, *Ole2*, and *Ole4*. Their transcripts were abundant and unique in developing seeds (MRE ranges between 10.55 and 276.3 relative to *β*-*actin*), with levels of expression significantly increased as Camelina seeds develop. Among oleosins, *Ole1* showed the maximum expression levels (~five-fold relative to the expression of both *Ole2* and *Ole4)*, while no significant difference observed in the expression levels between *Ole2* and *Ole4*. The seed-specific expression patterns observed for oleosins are consistent with their critical roles in determining the sizes and structures of oil bodies, thus controlling the number of TAG molecules accumulated and the mobilization of these molecules during seed germination [37]. In addition to the three well-characterized oleosins reported above, our transcriptome also revealed the detection of another twenty genes belong to oleosins, caleosins, and steroleosins (see Additional file 5: Table S5).

#### Triacylglycerol degradation

In *Arabidopsis*, many genes have been identified to be putative TAG lipases or fatty-acid de-esterifying lipases, which participate in lipid breakdown in different plant tissues [38]. The transcripts of many genes with potential lipase activities were detected in the Camelina transcriptome (Additional file 1: Table S9). These include genes similar to *ArabidopsisLip1* (At2g15230.1) and *SDP1* (AT5G04040.1), but with low expression levels. These two genes along with *SDP1L* gene were shown to direct the oil breakdown in the seeds during germination in *Arabidopsis* [38, 39]. However, their expression patterns observed here were inconsistent with the above findings of involvement in TAG degradation, and this suggests more candidates, that warrant further characterizations to understand the balance between TAG synthesis and degradation. In this regard, we identified another putative TAG lipase that belongs to *MGAT* protein family annotated as lysophospholipase 2 (*LysoPL2*) in *Arabidopsis* (At1g52760). The transcripts for *MGAT* showed ubiquitous expression patterns, with extensively increased levels of expression in developing seeds (Additional file 1: Table S9). This gene appears to maintain both MAG-acyltransferase activity and MAG/DAG hydrolase activity as reported by Vijayaraj et al. [31]. Its high levels of expression probably contribute to these activities, thus prove as an important new candidate gene for further understanding TAG biosynthesis.

Collectively, the gene-expression profiling of the candidate genes involved in TAG biosynthesis, illustrated in Additional file 1: Table S9 and the model shown in Fig. 7, highlighted the importance of those genes in the metabolic pathways of oil synthesis and accumulation in Camelina seeds. Further characterization of these genes will shed light on identifying the rate-limiting steps for TAG metabolism pathways in Camelina similar to other oilseed crops.

### Validation of transcript abundance using qRT-PCR

**Fig. 8:**
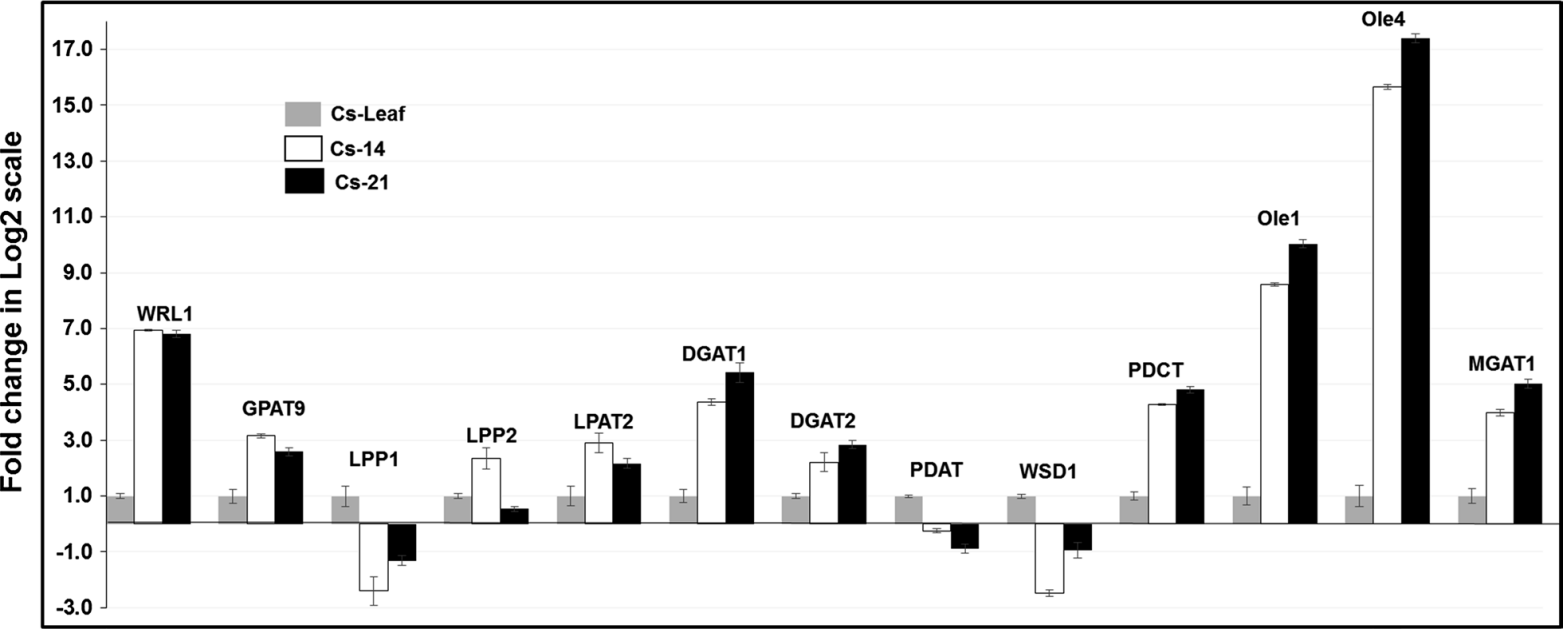
Expression of TAG biosynthesis-associated genes in Camelina developing seeds and leaf measured by qRT-PCR. Relative combined expression of all three copies of CsWRl1, CsGPAT9, CsLPP1, CsLPP2, CsLPAT2, CsDGAT1, CsDGAT2, CsPDAT, CsWSD1, CsPDCT, CsOle1, CsOle4, and CsMGAT1. The descriptive gene names are available in supplemental tables. The *bars* represent the fold change in log2 scale as measured by real-time qPCR from cDNA derived from Camelina seeds at 10–15 (Cs-14) and 16–21 (Cs-21) days after flowering (DAF) and from leaf tissue (Cs-Leaf). The leaf sample was used as the calibrator for the remaining samples. *Error bars* represent the standard error ± SE of three biological replicates. The quantification of the genes is measured relative to the expression of the indigenous housekeeping gene β-actin

To validate the quantification of the levels of Camelina genes obtained from RNA-Seq approach, the relative gene expressions of 13 TAG-related candidate genes were measured by qRT-PCR (Fig. 8). As shown in the presented data, there is a strong agreement between the results of qPCR and RNA-Seq for the 13 candidate genes examined. Within developing seeds, no significant difference in the expression abundance was observed between Cs-14 and Cs-21 for almost all the genes, while in seed-to-leaf comparisons, many genes showed to be differentially regulated. The genes, including *WRl1*, *GPAT9*, *LPAT2*, *DGAT1*, *DGAT2*, *PDCT*, *Ole1*, *Ole4*, *MGAT1*, and *LPP2* (lipid phosphate phosphatase 2). are relatively up-regulated in seeds compared to leaf, whereas the expression of *PDAT1*, *WSD1*, and LPP1 ((lipid phosphate phosphatase 1) genes was shown to be down-regulated. During seed development, the genes *WRI1*, *GPAT9*, *LPP1*, LPP2, *LPAT2*, and *WSD1* were shown to be up-regulated at the early seed stage, whereas the expression of *DGAT1*, *DGAT2*, *PDAT*, *PDCT*, *Ole1*, *Ole4*, and *MGAT1* genes peaked at the late seed stage.

Overall, the fold changes in the difference of gene expression estimated for the 13 genes (Additional file 1: Table S10, Fig. S3) showed positive correlation within developing seeds (r = 0.77, Additional file 1: Fig. S2A) and between seeds and leaf comparisons (r = 0.83, Additional file 2: Figure S2B (r = 0.87), Fig. S2C). As expected, discrepancies were the largest among the genes expressed at low levels (i.e., *LPP1*, *LPP2*, *LPAT*, and *WSD1*), which could be the reason for the decreased degree of correlation of those genes expressions, in addition to the variability in the dynamic range of the RNA-Seq and qPCR data scales. Moreover, because of the polyploidy nature of Camelina genome, there are at least more than one copy of each gene, and this itself a challenge to detect the expression of a single-gene copy using the accessible and limited routines included in the RNA-Seq data analysis. Therefore, the PCR primers combinations used were designed to detect all three copies of each gene. Accordingly, the expression abundance measured here is the aggregate expression for all duplicates identified for each gene, and thus ultimately, we have compared a single-expression value obtained by qPCR for each gene to the corresponding three values obtained by RNA-Seq for that particular gene. All of these sources of variation discussed above appear to significantly affect the correlation between the expression data detected by both qRT-PCR and RNA-Seq techniques.

## Conclusions

Next-generation RNA sequencing was performed on mRNAs obtained from Camelina developing seeds. Overall, the genome-guided assembly used here has significantly increased both the depth and the coverage of the analysis, thus widely identifying the genes and gene networks involved in various metabolic pathways controlling the amount and composition of oil accumulated in Camelina seeds. Our analysis focused on identifying the key factors in fatty-acid synthesis, TAG assembly and accumulation, and TAG degradation. Many of the identified transcripts showed temporal and differential expression patterns during Camelina seed development, and were involved in various functional categories to ultimately provide the metabolite intermediates which are utilized in lipid synthesis and storage.

Furthermore, the tissue-specific gene expression was profiled using RNA-Seq and was confirmed by qRT-PCR. The results of which were consistent regarding the expression patterns and the tissue specificity of the selected genes.

Since our analysis was concentrated on identifying the rate-limiting step(s) in oil synthesis in Camelina, we assessed the expression patterns and highlighted the transcripts involved in the formation of acyl chains in plastids. The highlighted transcripts were shown to be required to determine the rate of fatty acids supply provided to the subsequent pathways of TAG assembly which, in return, occurred mainly in the ER. This activity of pushing the fatty acids into the cytoplasm appears to be regulated by the activity of the transcription factor WRl1—which shows reasonable extensive and exclusive expression abundance in Camelina seeds. The aforementioned activity should be integrated with a pulling activity which is controlled by the enzymes settled in the ER. They, in turn, utilize the synthesized pools of acyl CoAs in various metabolic routes toward TAG synthesis and accumulation. In this regard, the enzymes controlling the synthesis of DAG, which ultimately limits the amount of TAG, which can be accumulated, were highlighted. The high and seed-specific expression levels detected for the genes *PAH1*, *DGAT1*, *PDCT*, *AAPT*, and *MGAT* support their direct involvement in DAG and TAG synthesis into Camelina seeds, and can be considered the rate-limiting factors in TAG metabolism; however, extended deep researches are required to confirm these findings.

Finally, the lack of well-characterized Camelina genome and the polyploidy nature of its genome for identifying the target genes for further analysis is very challenging and requires much more sophisticated analysis, including transcriptome integrated into metabolome and proteome approaches. This integration of knowledge would lead to develop research programs focus on engineering Camelina varieties with improved seed and oil qualities, which could better fit the various industrial applications suggested for Camelina oils.

## Methods

### Plant material

*Camelina sativa* cultivar ‘Suneson’ was grown in the greenhouse at 22 °C under natural light conditions supplemented with high-pressure sodium lights with a 16 h photoperiod (16 h of light and 8 h of darkness). The plants were watered and were fertilized with 200 ppm N of Peters professional 20-10-20 peat-lite special on a regular basis. During inflorescence, the emerging flowers were tagged, and flowers and seed pods at 7, 14, 21, and 28 days after flowering (DAF) were harvested to be used for quantitative real-time PCR (qRT-PCR), while seed pods at 10–15 DAF (Cs-14) and 16–21 DAF (Cs-21) were harvested for RNA sequencing. Young leaves were harvested from plants grown in half-strength Murashige and Skoog (MS) medium (PhytoTechnology Laboratories, Shawnee Mission, KS). The harvested Camelina tissues were immediately chilled in liquid nitrogen and kept frozen in −80 °C till RNA extraction.

### Fatty-acid composition and oil content determination

Fatty-acid methyl esters (FAME) were prepared from Camelina developing and mature seeds according to the method described in Li et al. [19]. Briefly, 25 mg Camelina seeds were freeze-dried for 48 h under high vacuum. The seed weight was recorded and the water content was determined as the weight difference of the seeds before and after freeze-drying. The seed tissues were triturated in 1.5 ml of toluene using a polytron, and then, a volume of 1 ml of 5 % (v/v) H_2_SO_4_ in MeOH, 25 μl of 0.2 % butylatedhydroxytoluene (BHT), and 5 µl of 10 mg/ml heptadecanoic acid (TAG 17:0) as an internal standard were added to the homogenate for the transmethylation reaction. The resulting residues were vortexed for 30 s and then heated at 95 °C for 90 min. Once cooled, a volume of 1.5 ml of 0.9 % NaCl and 2 ml hexane was then added to the homogenate and the phases were separated by centrifugation at 2000 rpm for 5 min. The upper organic phase was transferred to a fresh tube, while the resulting aqueous phase was extracted with a volume of 2 ml hexane and the phases were separated by centrifugation under the same conditions. The organic phases were pooled, and the solvent was removed by drying the samples under a flow of N_2_ gas. A volume of 5 ml hexane was then added to each sample and 1 ml was transferred to gas chromatography (GC) vials. The extracted FAMEs were analyzed on GC–MS-QP2010 SE (Shimadzu) in triplicate (from three biological replicates). The fatty acids were identified by comparison of their retention times with those of known standards, and the results were confirmed by searching the NIST mass spectrum library. The oil content was quantified by comparing the concentration of the fatty acids to the peak areas of the internal standard of known concentration.

### RNA extraction and cDNA library preparation

For the qRT-PCR studies, total RNA was isolated using plant RNeasy mini kit (Sigma-Aldrich) according to the manufacturer’s procedure. RNA concentrations were measured in ng/µl, and purity ratios (260/280 nm and 260/230 nm) were calculated using NanoDrop 2000 spectrophotometer (Thermo Scientific). A 1 µg RNA of each sample was used for the first strand cDNA synthesis using Verso cDNA Synthesis Kit (Thermo Scientific) in a cycling program of 42 °C for 30 min in one cycle following manufacturer’s instructions. The cDNA pools were quantified and then diluted to a final concentration of 100 ng/ul and were used as templates for qRT-PCR.

For RNA sequencing, total RNAs were extracted from Camelina developing seeds and were purified and quantified as described above. Three RNA preparations (biological replicates) for each sample were used to increase the sequencing coverage. Purified 5 µg total RNA for each sample was shipped to the RTSF Genomics Core at Michigan State University for cDNA libraries preparation and RNA sequencing.

### Quantitative real-time PCR (qRT-PCR)

All qRT-PCR reactions were performed in Eppendorf Mastercycler^®^ep realplex thermal cycler using the intercalation dye ABsolute Blue QPCR SYBR Green master mix kit (Thermo Scientific) as a fluorescent reporter. All PCR reactions were performed in triplicates for three biological replicates in 25 µl volumes using 1 µl of each forward and reverse primers (25 pmol each), 12.5 µl of SYBR green master mix, 1 µl of cDNA (100 ng/µl), and 9.5 µl HPLC molecular biology grade water. The cDNAs were amplified, and PCR products were quantified, using gene-specific primers, in the qPCR cycling program of 1 cycle at 95 °C for 15 min, 30–40 cycles at 95 °C for 15 s, 50–60 °C for 30 s, and 72 °C for 30 s. The quantification of PCR products was performed using the 2-ΔΔCt method [40], and the Camelina β-actin gene was used as internal reference to normalize the relative amount of mRNAs for all samples. The error bars represent the standard errors for the fold changes of relative gene expression calculated from two independent biological replicates and triplicate PCR reactions for each sample. Flowers and leaf samples were used here as controls. The PCR primers for candidate genes quantified by qRT-PCR are summarized in Additional file 1: Table S1.

### Library preparation for Illumina sequencing

RNA samples were prepared for sequencing using the Illumina TruSeq stranded mRNA library preparation kit LT. After quality control (QC), the libraries were combined and loaded on one HiSeq 2500 rapid run flow cell (v1); a flow cell contains two lanes. Sequencing was performed on an Illumina HiSeq 2500 using standard Rapid SBS reagents and procedures. As a result, for each sample, two sets of pair-end read files were generated, one from each lane. Base calling was done with Illumina real-time analysis (RTA) software (v1.17.21.3). Reads were demultiplexed, converted to FASTQ files by the Illumina Bcl2Fastq software (v1.8.4), and the FASTQ files were created.

### Data filtering and reads mapping against a reference genome

The raw RNA sequencing reads were trimmed by passing through quality control QC filters to remove adapter sequences, low-quality sequence (score >0.05), ambiguous nucleotides Ns, and terminal nucleotides in both 3′ and 5′ ends, as well as relatively short reads (<40 bp). The trimmed sequences were then analyzed using CLC Genomics Workbench 7.5 (http://www.clcbio.com). To identify the transcriptome content and quantify transcript abundance, the trimmed sequences were aligned against Camelina reference genome (*Cs_genome_sequence_build_V2.0*) which contain a total of 89,418 genes and 94,495 transcripts, as annotated by Kagale and his coworkers in 2014 [41] in the Prairie Gold project (http://www.camelinadb.ca). The alignment to Camelina reference genome was performed for each of the three biological replicates individually, and both genes and mRNA annotations in that genome were used for mapping and annotation. The alignment parameters were optimized to increase the mapping coverage and depth.

### Transcripts abundance and differential expression analysis

The short sequence reads were mapped to the genes and transcripts assigned into the reference genome according to the method developed by Mortazavi and his coworkers in 2008 [42]. The expression values for each Camelina transcript were estimated by applying within-library normalization by dividing the summarized read counts by the length of the gene and normalizing by ‘mapped reads’ which is known as RPKM (reads per kilobase of exon model per million mapped reads). The correlation coefficient (r) among the technical replicates for each sample was calculated, and the data showed strong positive correlation (R2 > 0.95) in all samples, supporting the idea of reproducibility of the RNA-Seq method. For the statistical analysis, the original RPKM values were quantile normalized, and then, a factor of 1 was added to them, and further, the RPKM + 1 values were log2 transformed and then used for further analysis.

To identify the differentially expressed genes (DEGs), comparative transcriptome analysis was performed on a different group of samples, and the means of expression (in log2 RPKMs) were statistically analyzed based on Gaussian distribution. A *t* test was used to estimate the significance of the difference of gene-expression means between different groups and the two-sided *P* values were calculated. False discovery rate (FDR) values were calculated using the method of Benjamin and Hochberg [42], from the distribution of *t* test *P* values.

Fold changes in expression between comparable conditions (Cs-14 vs Cs-21, Cs-14 vs Cs-Leaf, and Cs-21 vs Cs-Leaf) were calculated using log2 RPKM + 1 ratio. Genes and transcripts were defined as differentially expressed if they passed the following filters: (1) RPKM + 1 log2 ratios were considered significant if ≥1.5 or ≤−1.5, (2) the significance (*P* value) was set to be ≤0.05, (3) FDR values ≤0.05, (4) RPKM ≥0.1, and (5) raw read count ≥10 reads. The DEGs that passed these filters were extracted and used for the further annotation analysis.

### Functional annotation and analysis of DEGs

The annotation of DEGs was performed using various bioinformatics tools involved in Blast2Go server (https://www.blast2go.com, [43]). The input sequences were compared against NCBI BLAST non-redundant database NR using BLAST X program (*E* value ≤1.0E−3). Hits with highest sequence similarities were retrieved, mapped, and gene ontology (GO) annotated, and the genes were distributed among distinct functional categories, including molecular function, cellular components, and biological process. Enzyme codes (ECs) were obtained by mapping from equivalent GOs, and structural motifs and domains were determined by applying InterPro Scan tool. Metabolite pathways in which the DEGs may involve were identified, and the relationships between GOs and ECs were highlighted on Kyoto encyclopedia of genes and genomes KEGG maps.

## Additional files

10.1186/s13068-016-0555-5 contains experimental results from total lipid determination analysis. **Fig. S2.** contains the Volcano plots for the relationship between fold change and P values for DEGs detection. **Fig. S3.** contains graphs for the comparisons between the qRT-PCR and RNA-Seq data analysis. **Table S1.** contains list of the selected TAG genes used for qRT-PCR and the designed PCR primers. **Table S8.** Top-10 differentially expressed genes in developing seeds and leaf tissues **Table S9.** contains list of the lipid metabolism-related genes with their expression levels. **Table S10.** contains comparison of the fold changes in gene expressions obtained using RNA-Seq and qRT-PCR methods. **Table S11.**. contains the expression abundance of TAG-related genes in relative to the housekeeping gene β-actin.
10.1186/s13068-016-0555-5 List of Camelina genes with the highest RPKM values.
10.1186/s13068-016-0555-5 List of the whole Camelina genes expressed in seed and leaf tissues.
10.1186/s13068-016-0555-5 Results of the comparative transcriptome analysis.
10.1186/s13068-016-0555-5 List of Camelina genes involved in lipid metabolism.
10.1186/s13068-016-0555-5 GO information for the DEGs differentially regulated in Cs-14.
10.1186/s13068-016-0555-5 GO information for the DEGs differentially regulated in Cs-21.

## Abbreviations

TAG: triacylglycerol
DAF: days after flowering
RPKM: reads per kilobase of transcript per million mapped reads
FAME: fatty-acid methyl ester
MUFA: monounsaturated fatty acids
PUFA: polyunsaturated fatty acids
LCFA: long-chain fatty acids
qRT-PCR: quantitative real-time polymerase chain reaction
RNA-seq: RNA sequencing
G3P: glycerol-3-phosphate
LPA: lysophosphatidic acid
PA: phosphatidic acid
DAG: diacylglycerol
DGAT: diacylglycerol acyltransferases
PDAT: phospholipid:diacylglycerol acyltransferases
GPAT: glycerol-3-phosphate acyltransferase
DEGs: differentially expressed genes
FDR: false discovery rate
BLAST: basic local alignment search tool
LPAT: lysophosphatidyl acyltransferase
MGAT: monoacylglycerol acyltransferase
PDCT: phosphatidylcholine:diacylglycerol cholinephosphotransferase
WRl1: wrinkled 1
WSD1: wax synthase:diacylglycerol acyltransferase
UCE10: ubiquitin-conjugating enzyme
RuBisCO: ribulose-1,5-bisphosphate carboxylase/oxygenase
GO: gene ontologies
KEGG: kyoto encyclopedia of genes and genomes
ER: endoplasmic reticulum
MRE: maximum relative expression
ACP: acyl-carrier protein
FFA: free fatty acids
LPC: lysophosphatidylcholine
PDH- E1-α E1-β: pyruvate dehydrogenase
PDC: pyruvate dehydrogenase complex
ACC1: acetyl-CoA carboxylase
ACCase-α: acetyl-CoA carboxylase, a-carboxyltransferase
KAS: ketoacyl-ACP synthase
ENR1: enoyl-ACP reductase
FATA: fata acyl-ACP thioesterase
SAD: stearoyl-ACP desaturase
LACS: long-chain acyl-CoA synthase
SDP1: sugar-dependent protein
SDP1-L: sugar-dependent 1-like protein
PAH: phosphatidic acid phosphohydrolase
DGK: diacylglycerol kinase
AGK: acylglycerol kinase
AAPT: aminoalcoholphosphotransferase
LPCAT: lysophosphatidylcholine acyltransferase
PLA2: phospholipase A2
Ole: oleosin
