# Supplementary material for: Transcriptome profiling of Camelina sativa to identify genes involved in triacylglycerol biosynthesis and accumulation in the developing seeds
Source: Biotechnol Biofuels. 2016 Jul 4;9:136. doi: 10.1186/s13068-016-0555-5 (PMC4932711; doi:10.1186/s13068-016-0555-5)
Supplement: Supplementary file 1 — 10.1186/s13068-016-0555-5 contains experimental results from total lipid determination analysis. Fig. S2. contains the Volcano plots for the relationship between fold change and P values for DEGs detection. Fig. S3. contains graphs for the comparisons between the qRT-PCR and RNA-Seq data analysis. Table S1. contains list of the selected TAG genes used for qRT-PCR and the designed PCR primers. Table S8. Top-10 differentially expressed genes in developing seeds and leaf tissues Table S9. contains list of the lipid metabolism-related genes with their expression levels. Table S10. contains comparison of the fold changes in gene expressions obtained using RNA-Seq and qRT-PCR methods. Table S11.. contains the expression abundance of TAG-related genes in relative to the housekeeping gene β-actin. [file 13068_2016_555_MOESM1_ESM.docx]

**Transcriptome Profiling of *Camelina sativa* to Identify Genes Involved in Triacylglycerol Biosynthesis and Accumulation in the Developing Seeds**

# Hesham M. Abdullah^1,2^, Parisa Akbari^1^, Bibin Paulose^3^, Danny Schnell^3^, Weipeng Qi^4^,

# Yeonhwa Park^4^, Ashwani Pareek^5^, and Om Parkash Dhankher^1*^

^1^Stockbridge School of Agriculture, University of Massachusetts Amherst, MA 01003, USA

^2^Biotechnology Department, Faculty of Agriculture, Al-Azhar University, Cairo 11651, Egypt

^3^Department of Plant Biology, Michigan State University, East Lansing, MI 48824, USA

^4^Department of Food Science, University of Massachusetts Amherst, MA 01003, USA

^5^Stress Physiology and Molecular Biology Laboratory, School of Life Science, Jawaharlal Nehru University, New Delhi 100067, India

^*^**Corresponding Author:** Om Parkash Dhankher; Email: [parkash@umass.edu](mailto:parkash@umass.edu)

**emails**Hesham M. Abdullah: [habdullah@psis.umass.edu](mailto:habdullah@psis.umass.edu)

Danny Schnell: [dschnell@cns.msu.edu](mailto:dschnell@cns.msu.edu)

Bibin Paulose: [bpaulose@msu.edu](mailto:bpaulose@msu.edu)

Ashwani Pareek: ashwanip@mail.jnu.ac.in

Parisa Akbari: pakbari@psis.umass.edu

Yeonhwa Park: [ypark@foodsci.umass.edu](mailto:ypark@foodsci.umass.edu)

Weipeng Qi: [wqi@umass.edu](mailto:wqi@umass.edu)


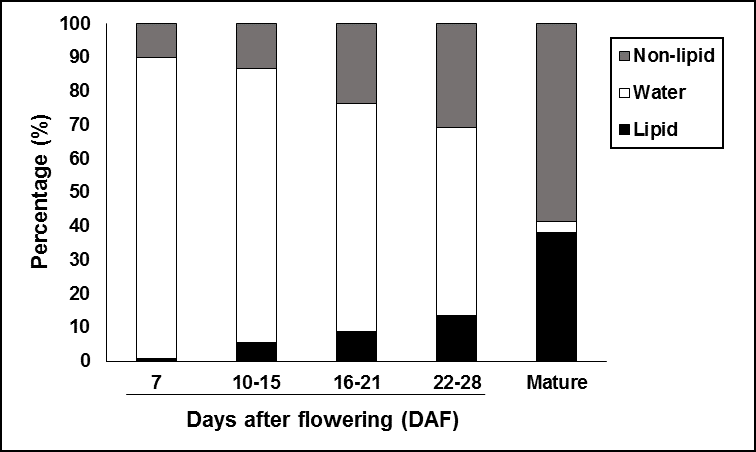


Fig S1. The changes (%) in the total lipids (black), water content (white), and non-lipid components (dark gray) in Camelina sativa during seed development. Data represent the mean of three independent measurements. Non-lipid components, accounts for proteins, sugars, and others.


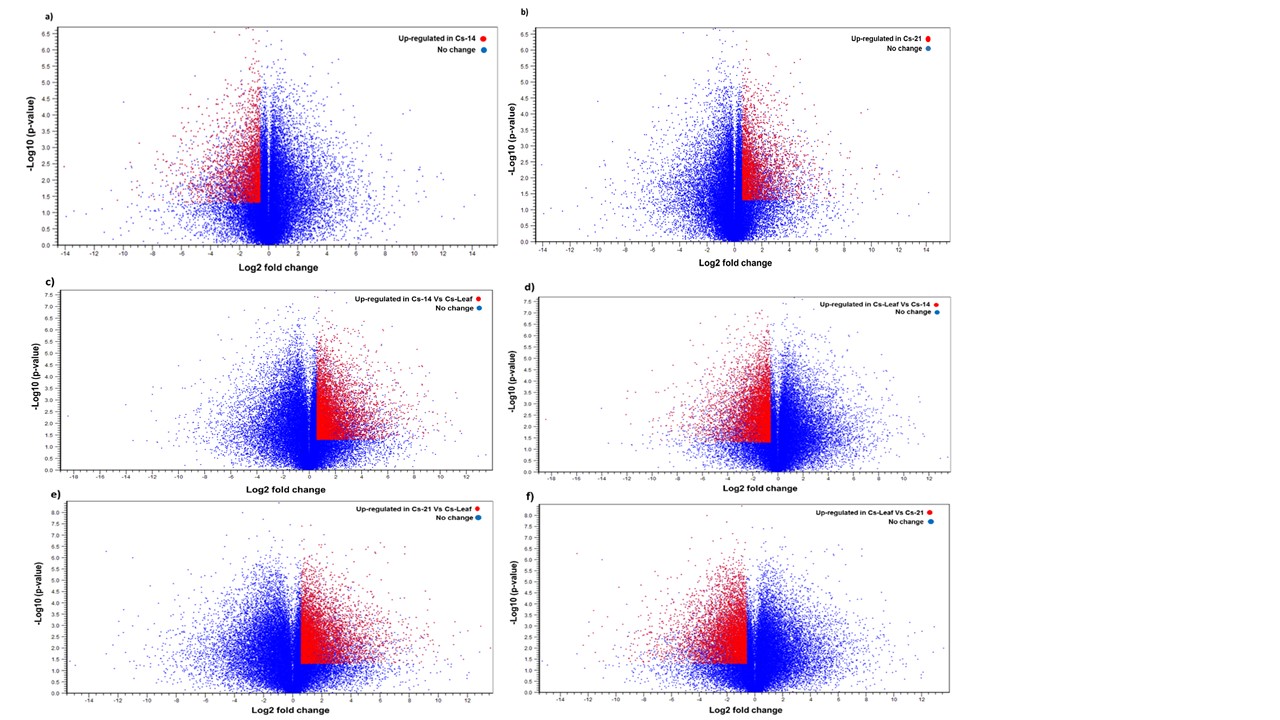


**Figure S2**. Volcano plot of the relationship between the P-value of statistical test (y-axis) and the log2 fold change (x-axis) showing the differentially expressed genes between different camelina tissues. The genes that showed significant difference (P-value ≤ 0.05) and fold change (log2 fold change ≥ 1.5 or ≤ -1.5) were presented as red spots. The number of up-regulated and down-regulated genes between Cs-14 and Cs-21, Cs-14 and Cs-Leaf, Cs-21 and Cs-Leaf are summarized. Between Cs-14 (10-15 DAF) and Cs-21 (16-21 DAF) camelina developing seeds, there are 3709 genes up-regulated in Cs-14 (a) and 4223 genes up-regulated in Cs-21 (b). Between Cs-14 and Cs-Leaf, there are 8676 genes up-regulated in Cs-14 (c) and 10737 genes up-regulated in Cs-Leaf (d). Between Cs-21 and Cs-Leaf, there are 9356 gens up-regulated in Cs-21 (e) and 10773 genes up-regulated in Cs-Leaf (f). In Cs-14 Vs Cs-21 comparison, Cs-14 was used as a reference, while Cs-Leaf was used as a reference in Cs-14 Vs Cs-Leaf and in Cs-21 Vs Cs-Leaf comparisons.


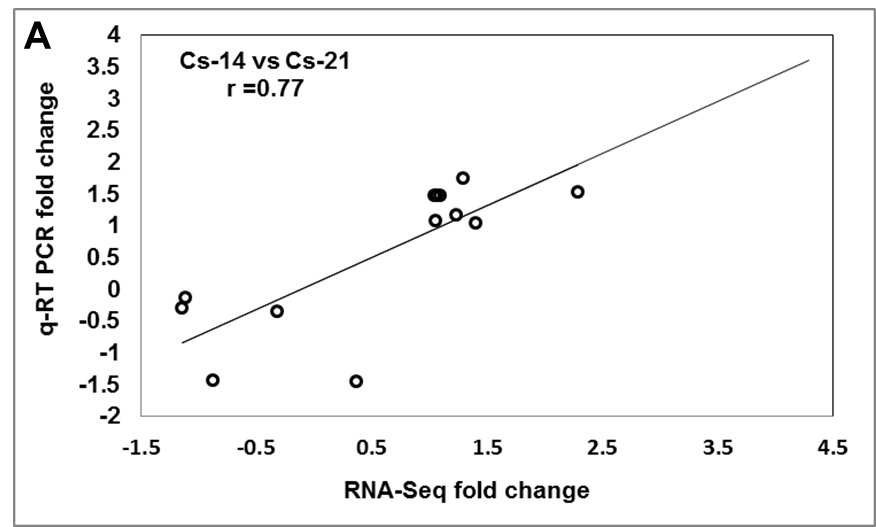

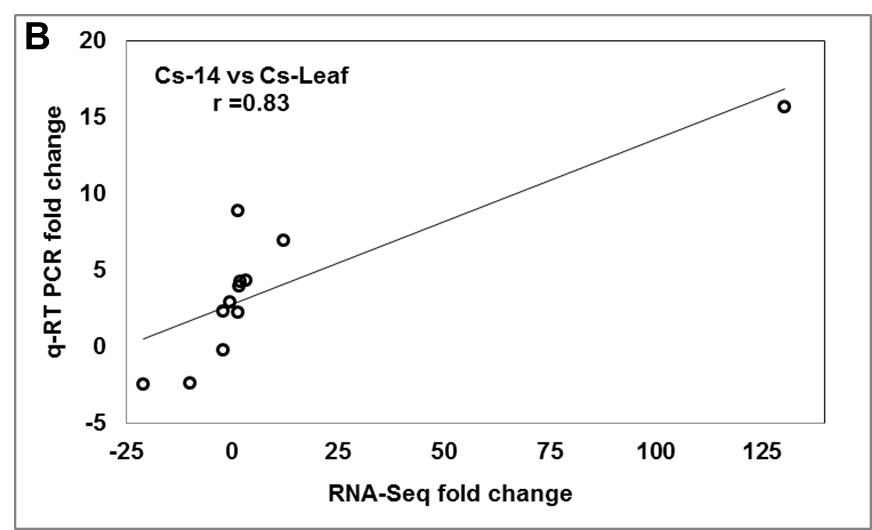

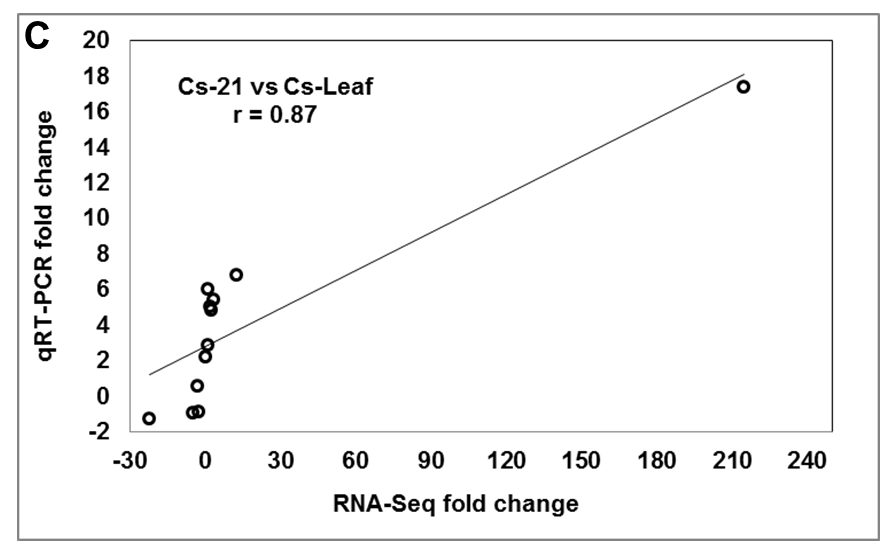


**Figure S3**. A comparison of fold change (in log2 scale) values of transcription levels for 13 genes measured by RNA-Seq and qRT-PCR. Fold change (log2) values for individual transcripts between two experimental conditions in which the mRNA levels were quantified by qRT-PCR analyses (y-axis), and then compared with corresponding values obtained by RNA-Seq experiments (x-axis). The pairs of experimental conditions compared include Camelina developing seeds; Cs-14 (10-15 DAF) and Cs-21 (16 -21 DAF) (A), Cs-14 and Cs-Leaf (B), and Cs-21 and Cs-Leaf (C). The values in RNA-Seq analysis for individual genes is the mean value for 3 gene copies obtained from camelina genome. r represent the correlation coefficient.

| Table S1. List of TAG genes used in qRT-PCR analysis. gene names, accession numbers, primer sequences, and size of amplification products are shown. | | | | |
| --- | --- | --- | --- | --- |
| Gene name | Gene ID | Gene Description | Forward/Reverse primers | Amplicon size |
| CsWRl1 | Csa06g028810.1  Csa04g040400.1  Csa09g064030.1 | WRINKLED1, encodes transcription factor of the AP2/ERWEBP, Integrase-type DNA-binding superfamily protein | 5’AGTATCCCGAGCTTTTCAACG3’ 5’GAAAAGCAAGACAACGGAGAAG3’ | 166 bp |
| CsGPAT9 | Csa02g067560.1  Csa11g094450.1  Csa18g033950.1 | glycerol-3-phosphate acyltransferase 9 | 5’AGGGATTGAGTTTGCCGAG3’  5’AATCTTGCCAGGATCGACTC3’ | 156 bp |
| CsLPAAT2 | Csa06g033410.1  Csa06g033390.1  Csa06g033430.1 | lysophosphatidyl acyltransferase 2 | 5’TGACTTTCCTCGACCTTTCTG3’  5’TGGCTGGGACAAATGAACG3’ | 185 bp |
| CsLPP1 | Csa09g001150.1  Csa06g001110.1  Csa04g002120.1 | phosphatidic acid phosphatase 1 | 5’AAATCAGGTGAGGTCAAGGAAG3’ 5’CATCCACACGAGATATCCCAAC3’ | 199 bp |
| CsLPP2 | Csa14g018220.1  Csa03g018880.1  Csa17g020640.1 | phosphatidic acid phosphatase 2 | 5’TTGGTGTATCCCGAGTTGATG3’  5’AGCATCTGGAAGTACGCATG3’ | 157 bp |
| CsPDAT | Csa13g016300.1  Csa08g005560.1  Csa20g019000.1 | PDAT1 phospholipid:diacylglycerol acyltransferase | 5’GATGACCCCAAGTATAAAGATCCC3’  5’TCGTGAGCAGAAGTGAAGATC3’ | 188 bp |
| CsDGAT1 | Csa01g042590.1  Csa19g056370.2  Csa15g084220.1 | membrane bound O-acyl transferase (MBOAT) family protein | 5’AAGATACCAAAGACACTCGCC3’  5’TGAGCCGAACCTTTCTTGTAG3’ | 171 bp |
| CsDGAT2 | Csa04g037310.1  Csa09g058550.1  Csa06g025650.1 | diacylglycerol acyltransferase family | 5’AGAACGTCTTCCTTTCATCGAG3’  5’AAGCAGATCGGAGTAAACCTG3’ | 172 bp |
| CsPDCT | Csa19g022610.1  Csa15g020460.1  Csa01g018440.1 | phosphatidic acid phosphatase-related / PAP2-related | 5’CATGGTTAGTGGAAGGACGAG3’  5’TCCCACCGGAAAATCAACTC3’ | 137 bp |
| CsMGAT | Csa17g092850.1  Csa03g059790.1  Csa17g092830.1 | lysophospholipase 2 | 5’GACAGCGGATGGAGTATCATG3’  5’CCATACCTCTTGACCCTTTCG3’ | 189 bp |
| CsOle 1 | [Csa12g028090.1](http://camelinadb.ca/prairiegold/cgi-bin/do_blast.cgi#34889)  [Csa11g019460.1](http://camelinadb.ca/prairiegold/cgi-bin/do_blast.cgi#5956)  [Csa10g017840.1](http://camelinadb.ca/prairiegold/cgi-bin/do_blast.cgi#69354) | Oleosin 1 | 5’GTTGGGAGAGGAAGATAAACCG3’  5’GTAGAAGTACTTGGGCCGTAAC3’ | 195 bp |
| CsOle 4 | Csa04g015780.1  Csa09g014800.1  Csa06g008780.1 | Oleosin 4 | 5’CTCTGTCTCGTGGGTCTTAAAC3’  5’AGCCTCATGTGCCTTATCTTG3’ | 157 bp |
| CsActin 2 | Csa15g026420_1  Csa19g026200_1  Csa01g021690_1 | β-Actin | 5’ACAATTTCCCGCTCTGCTGTTGTG3’ 5’AGGGTTTCTCTCTTCCACATGCCA3’ | 220 bp |

| Table S8. Top-10 differentially expressed genes in developing seeds and leaf tissues. | | | | | | |
| --- | --- | --- | --- | --- | --- | --- |
| Gene ID | **Camelina designate** | | **Cs-14 RPKM** | **Cs-21 RPKM** | **Log2 fold change** | **P-value** |
|  |  | | **Cs-14 Vs Cs-21** | | | |
| High abundance in Cs-14 | | | | | | |
| Csa09g096810_1 | unknown protein | | 13.480 | 8.109 | -1.66 | 1.69E-02 |
| Csa09g075850_1 | Kunitz family trypsin and protease inhibitor protein | | 12.122 | 7.573 | -1.60 | 4.60E-03 |
| Csa16g054910_1 | unknown protein | | 11.584 | 7.414 | -1.56 | 3.05E-05 |
| Csa09g075860_1 | Kunitz family trypsin and protease inhibitor protein | | 11.420 | 7.087 | -1.61 | 6.76E-04 |
| Csa18g032610_1 | lipid transfer protein 4 | | 10.962 | 6.358 | -1.72 | 6.14E-05 |
| Csa07g063460_1 | unknown protein | | 10.939 | 4.933 | -2.22 | 2.76E-06 |
| Csa11g093140_1 | lipid transfer protein 4 | | 10.434 | 2.853 | -3.66 | 5.24E-05 |
| Csa09g075840_1 | Kunitz family trypsin and protease inhibitor protein | | 9.666 | 3.802 | -2.54 | 1.82E-03 |
| Csa02g065010_1 | lipid transfer protein 4 | | 9.591 | 1.382 | -6.94 | 4.20E-04 |
| Csa11g093150_1 | lipid transfer protein 4 | | 9.291 | 1.449 | -6.41 | 3.34E-05 |
|  | Others | |  |  |  |  |
| High abundance in Cs-21 | | | | | | |
| Csa20g077530_1 | Protein of unknown function (DUF1264) | | 5.891 | 8.868 | 1.51 | 6.47E-03 |
| Csa18g005520_1 | Protein of unknown function (DUF1264) | | 5.590 | 8.707 | 1.56 | 4.01E-03 |
| Csa02g076390_1 | Dehydrin family protein | | 5.419 | 8.447 | 1.56 | 1.60E-02 |
| Csa17g007790_1 | AWPM-19-like family protein | | 5.527 | 8.421 | 1.52 | 8.42E-03 |
| Csa04g044980_1 | Adenine nucleotide alpha hydrolases-like superfamily protein | | 5.085 | 8.243 | 1.62 | 7.53E-03 |
| Csa12g037540_1 | Late embryogenesis abundant protein (LEA) family protein | | 5.236 | 8.227 | 1.57 | 3.22E-03 |
| Csa02g033690_1 | Tetratricopeptide repeat (TPR)-like superfamily protein | | 5.181 | 8.155 | 1.57 | 8.28E-03 |
| Csa09g068650_1 | Iron/manganese superoxide dismutase family protein | | 5.100 | 8.060 | 1.58 | 6.21E-03 |
| Csa16g043300_1 | dehydrin LEA | | 5.185 | 7.945 | 1.53 | 6.90E-03 |
| Csa10g028690_1 | spermidine disinapoyl acyltransferase | | 5.064 | 7.899 | 1.56 | 4.90E-03 |
|  | Others | |  |  |  |  |
|  |  | | **Cs-14 Vs Cs-Leaf** | | | |
| High abundance in Cs-14 | | | | | | |
| Csa18g032610_1 | lipid transfer protein 4 | 11.109 | 2.373 | 4.68 | 3.80E-06 | |
| Csa04g061240_1 | Scorpion toxin-like knottin superfamily protein | 10.307 | 6.542 | 1.58 | 3.87E-03 | |
| Csa05g006880_1 | Scorpion toxin-like knottin superfamily protein | 10.269 | 3.342 | 3.07 | 3.77E-03 | |
| Csa10g042420_1 | Oleosin family protein | 10.144 | 0.513 | 19.78 | 1.51E-04 | |
| Csa09g097100_1 | Vacuolar calcium-binding protein-related | 10.052 | 3.896 | 2.58 | 1.81E-03 | |
| Csa02g065010_1 | lipid transfer protein 4 | 9.909 | 2.036 | 4.87 | 3.39E-03 | |
| Csa11g024290_1 | Peroxidase superfamily protein | 9.827 | 5.496 | 1.79 | 1.61E-04 | |
| Csa19g001360_1 | Oleosin family protein | 9.778 | 0.559 | 17.50 | 1.19E-03 | |
| Csa11g093150_1 | lipid transfer protein 4 | 9.699 | 0.673 | 14.41 | 2.18E-03 | |
| Csa18g033140_1 | Gibberellin-regulated family protein | 9.696 | 1.479 | 6.55 | 4.65E-05 | |
|  | Others |  |  |  |  | |
| High abundance in Cs-Leaf | | | | | | |
| Csa11g053300_1 | Ribulose bisphosphate carboxylase (small chain) family protein | 1.812 | 12.796 | -7.06 | 5.50E-07 | |
| Csa12g084200_1 | Ribulose bisphosphate carboxylase (small chain) family protein | 0.227 | 11.616 | -51.28 | 2.02E-03 | |
| Csa12g084210_1 | Ribulose bisphosphate carboxylase (small chain) family protein | 1.373 | 11.527 | -8.39 | 7.31E-06 | |
| Csa10g044490_1 | Ribulose bisphosphate carboxylase (small chain) family protein | 0.586 | 11.417 | -19.48 | 1.17E-06 | |
| Csa04g053880_1 | rubisco activase | 4.694 | 11.403 | -2.43 | 6.50E-04 | |
| Csa07g032360_1 | ribulose bisphosphate carboxylase small chain 1A | 6.580 | 11.353 | -1.73 | 4.06E-03 | |
| Csa10g044480_1 | Ribulose bisphosphate carboxylase (small chain) family protein | 0.197 | 11.235 | -56.95 | 3.57E-03 | |
| Csa05g013300_1 | rubisco activase | 4.588 | 11.168 | -2.43 | 2.17E-05 | |
| Csa06g043830_1 | rubisco activase | 4.906 | 10.908 | -2.22 | 3.06E-04 | |
| Csa04g030500_1 | glycine-rich protein 3 | 1.728 | 10.524 | -6.09 | 5.84E-03 | |
|  | Others |  |  |  |  | |
|  |  | **Cs-21 Vs Cs-Leaf** | | | | |
| High abundance in Cs-21 | | | | | | |
| Csa04g061240_1 | Scorpion toxin-like knottin superfamily protein | 12.019 | 6.457 | 1.86 | 1.78E-03 | |
| Csa10g042420_1 | Oleosin family protein | 11.325 | 0.430 | 26.32 | 1.23E-04 | |
| Csa19g001360_1 | Oleosin family protein | 10.999 | 0.473 | 23.28 | 9.65E-04 | |
| Csa06g008780_1 | oleosin 4 | 10.340 | 0.430 | 24.05 | 7.93E-04 | |
| Csa04g061210_1 | Scorpion toxin-like knottin superfamily protein | 10.008 | 0.617 | 16.22 | 1.61E-03 | |
| Csa06g049790_1 | Scorpion toxin-like knottin superfamily protein | 9.893 | 1.111 | 8.90 | 5.33E-04 | |
| Csa06g049820_1 | Scorpion toxin-like knottin superfamily protein | 9.750 | 5.631 | 1.73 | 6.67E-03 | |
| Csa09g097100_1 | Vacuolar calcium-binding protein-related | 9.742 | 3.775 | 2.58 | 2.11E-03 | |
| Csa03g015710_1 | 2-oxoglutarate (2OG) and Fe(II)-dependent oxygenase superfamily protein | 9.720 | 0.191 | 50.77 | 1.84E-04 | |
| Csa20g077530_1 | Protein of unknown function (DUF1264) | 9.522 | 0.011 | 905.33 | 1.41E-04 | |
|  | Others |  |  |  |  | |
| High abundance in Cs-Leaf | | | | | | |
| Csa05g079910_1 | ribulose bisphosphate carboxylase small chain 1A | 8.463 | 14.122 | -1.67 | 3.45E-07 | |
| Csa11g053300_1 | Ribulose bisphosphate carboxylase (small chain) family protein | 0.871 | 13.828 | -15.88 | 4.15E-05 | |
| Csa16g026850_1 | ribulose bisphosphate carboxylase small chain 1A | 7.934 | 13.457 | -1.70 | 3.15E-03 | |
| Csa12g084210_1 | Ribulose bisphosphate carboxylase (small chain) family protein | 0.379 | 11.777 | -31.05 | 5.57E-04 | |
| Csa12g084200_1 | Ribulose bisphosphate carboxylase (small chain) family protein | 0.380 | 11.758 | -30.98 | 2.15E-05 | |
| Csa07g058110_1 | photosystem II subunit R | 7.611 | 11.589 | -1.52 | 4.20E-03 | |
| Csa10g044490_1 | Ribulose bisphosphate carboxylase (small chain) family protein | 0.173 | 11.551 | -66.93 | 3.48E-07 | |
| Csa04g053880_1 | rubisco activase | 3.078 | 11.546 | -3.75 | 3.45E-04 | |
| Csa07g032360_1 | ribulose bisphosphate carboxylase small chain 1A | 4.973 | 11.511 | -2.31 | 1.81E-03 | |
| Csa10g044480_1 | Ribulose bisphosphate carboxylase (small chain) family protein | 0.102 | 11.390 | -111.46 | 3.80E-04 | |
|  | Others |  |  |  |  | |
| Expression values of Camelina gene models and significant differential expression between camelina tissues. RPKM values assigned to each gene are shown. Log2 fold change (>1.5 or < -1.5) and P-Values (< 0.05) for each transcript are shown. The full list of the DEGs in each pairwise comparison presented here are listed in Table S4; Cs-14 Vs Cs-21, Cs-14 Vs Cs-Leaf, and Cs-21 Vs Cs-Leaf sheets. | | | | | | |

| Table S9. Expression quantification of selected genes related to Oil synthesis, accumulation, and breakdown in Camelina | | | | | | | | | | | | | | |
| --- | --- | --- | --- | --- | --- | --- | --- | --- | --- | --- | --- | --- | --- | --- |
| Camelina ID | **Arabidopsis Homologous** | | | | **Gene Description** | **Cs-14 RPKM** | | **Cs-21**  **RPKM** | **Cs-Leaf RPKM** | | **Log2 Fold change** | | | |
|  |  |  |  |  |  |  |  |  |  |  | **Cs-14 Vs Cs-21** | | **Cs-14 Vs Cs-leaf** | **Cs-21 Vs Cs-leaf** |
| Fatty acid Biosynthesis | | | | | | | | | | | | | | |
| Csa17g001570.1  Csa14g002090.1  Csa03g002150.1 | | | At4g33070 | Pyruvate dehydrogenase E1-α  PDH- E1-α | | | 83.77  77.02  75.94 | 51.55  48.40  61.83 | | 24.95  26.42  19.01 | -1.07  -1.11  -1.04 | 1.51  1.49  1.62 | | 1.41  1.33  1.56 |
| Csa17g043080.1  Csa14g037700.1  Csa03g033280.1 | | | At1g30120 | Pyruvate dehydrogenase E1-β PDH-E1-β | | | 83.36  47.83  20.95 | 54.17  30.05  14.22 | | 20.73  20.10  14.43 | -1.06  -1.08  -1.06 | 1.59  1.38  1.16 | | 1.50  1.27  1.07 |
| Csa03g038570.1  Csa14g044300.1  Csa17g059730.1 | | | At1g34430 | pyruvate dehydrogenase complex PDC | | | 23.59  39.13  27.73 | 9.31  19.70  14.79 | | 11.86  18.21  15.59 | -1.28  -1.15  -1.14 | 1.28  1.34  1.25 | | -1.05  1.14  1.07 |
| Csa15g052810.1  Csa01g031130.1  Csa19g037960.1 | | | At3g25110 | FatA Acyl-ACP Thioesterase ATFATA | | | 39.31  16.82  27.27 | 60.32  25.87  46.38 | | 1.88  1.15  2.19 | 1.16  1.21  1.21 | 3.04  2.96  2.51 | | 3.86  4.00  3.33 |
| Csa10g034800.1  Csa11g043130.2  Csa12g065690.1 | | | At4g13050 | Acyl-ACP Thioesterase FatA | | | 11.09  15.59  11.60 | 8.10  13.59  9.33 | | 4.99  4.07  5.26 | -1.04  1.02  -1.01 | 1.25  1.57  1.25 | | 1.20  1.65  1.26 |
| Csa17g011970.1  Csa14g009990.1  Csa03g011960.1 | | | At1g08510 | Fatty Acyl-ACP Thioesterase B FatB | | | 15.74  12.47  7.56 | 16.02  9.25  7.26 | | 11.98  14.09  10.01 | 1.07  -1.04  1.08 | 1.10  -1.06  -1.22 | | 1.20  -1.12  -1.13 |
| Csa14g047290.1  Csa03g039320.1  Csa05g058160.1 | | | At1g36160 | Acetyl-CoA carboxylase ACC1 | | | 2.83  3.11  2.83 | 2.04  2.80  1.89 | | 1.76  0.99  1.92 | -1.05  1.07  -1.09 | -1.08  1.36  -1.12 | | -1.15  1.52  -1.26 |
| Csa07g013000.1  Csa05g033570.1  Csa16g014700.1 | | | At2g30200.1 | Acyl-carrier-protein S-malonyltransferase EMP3147 | | | 46.83  31.82  30.47 | 20.95  15.67  13.00 | | 22.54  23.34  21.45 | -1.18  -1.17  -1.23 | 1.33  1.17  1.18 | | 1.10  -1.02  -1.07 |
| Csa04g051720.1  Csa05g015510.1  Csa06g040780.1 | | | At2g38040 | Acetyl-CoA carboxylase, a-carboxyltransferase Accase-α | | | 55.24  49.36  55.26 | 41.01  33.52  36.48 | | 11.36  10.56  9.38 | -1.03  -1.06  -1.06 | 1.69  1.68  1.79 | | 1.68  1.61  1.72 |
| Csa20g058900.1  Csa08g032230.1  Csa00630s010.1 | | | At2g05990.1 | Enoyl-ACP reductase ENR1 | | | 49.83  127.86  28.12 | 31.81  80.55  16.94 | | 35.08  38.12  18.55 | -1.08  -1.07  -1.10  -1.08  -1.08 | 1.21  1.50  1.20 | | 1.12  1.42  1.08 |
| Csa18g003740.1  Csa20g079430.1  Csa11g065710.1 | | | At5g46290 | Ketoacyl-ACP Synthase KASI | | | 96.60  66.88  61.98 | 60.34  54.55  37.41 | | 48.33  40.66  41.72 | -1.07  -1.00  -1.09 | 1.33  1.26  1.23 | | 1.24  1.27  1.13 |
| Csa16g038860.1  Csa07g046400.2  Csa09g079550.1 | | | At1g74960 | Ketoacyl-ACP Synthase KASII | | | 61.15  55.73  63.77 | 49.62  45.42  57.96 | | 14.91  10.48  18.29 | -1.00  -1.01  1.02 | 1.61  1.74  1.53 | | 1.63  1.77  1.59 |
| Csa16g055340.1  Csa09g097250.1  Csa07g063910.1 | | | At1g62640 | Ketoacyl-ACP Synthase KASIII | | | 27.11  28.01  3.22 | 19.46  20.39  2.90 | | 8.30  7.76  2.61 | -1.04  -1.04  1.08 | 1.50  1.55  -1.19 | | 1.45  1.51  -1.10 |
| Csa06g053760.1  Csa04g066390.3  Csa05g001930.1 | | | AT2G47240.1 | Long chain Acyl-CoA synthase LACS | | | 3.18  5.33  0.83 | 3,38  6.28  0.50 | | 5.12  1.91  1.47 | 1.17  1.18  -1.17 | -1.58  1.29  -2.36 | | -1.35  1.36  -2.90 |
| Csa06g050010.1  Csa04g061470.1  Csa05g006640.1 | | | AT2G43710.2 | Stearoyl-ACP desaturase SAD | | | 140.95  101.31  86.14 | 138.76  102.10  97.57 | | 47.06  31.42  37.58 | 1.02  1.03  1.06 | 1.46  1.49  1.37 | | 1.51  1.57  1.48 |
| Csa06g028810.1  Csa09g064030.2  Csa04g040400.2 | | | [AT3G54320.1](http://arabidopsis.org/servlets/TairObject?type=gene&id=40775) | Wrinkled 1 WRl1 | | | 53.82  44.95  31.69 | 30.01  27.98  17.23 | | 0.38  0.27  0.16 | -1.11  -1.09  -1.14 | 9.17  11.56  15.84 | | 9.34  12.27  16.14 |
| Triacylglycerol Biosynthesis | | | | | | | | | | | | | | |
| Csa09g094530.1  Csa07g059410.1  Csa16g049970.1 | | | AT1G80460.1 | Protein-similar to Glycerol Kinase NHO1 | | | 4.98  2.65  5.62 | 3.15  1.21  3.58 | | 3.82  3.65  11.0 | -1.11  -1.39  -1.11 | -1.08  -1.55  -1.46 | | -1.25  -2.35  -1.71 |
| Csa03g009940.1  Csa14g007930.1  Csa17g009910.1 | | | AT1G06520 | glycerol-3-phosphate acyltransferase GPAT1 | | | 1.75  0.30  0.72 | 0.81  0.69  1.22 | | 1.16  0.77  3.65 | -1.41  2.58  1.82 | -1.19  -3.82  -4.07 | | -1.77  -1.51  -2.34 |
| Csa03g002410.1  Csa14g002450.1  Csa17g001170.1 | | | AT1G02390 | glycerol-3-phosphate acyltransferase GPAT2 | | | 2.15  3.40  2.77 | 0.58  1.62  1.41 | | 0.09  0.36  0.07 | -2.06  -1.33  -1.30 | 7.04  2.93  11.19 | | 3.52  2.26  9.57 |
| Csa02g005090.1  Csa08g052570.1  Csa13g055330.1 | | | AT4G01950 | glycerol-3-phosphate acyltransferase GPAT3 | | | 0.05  0.46  0.04 | 0.01  0.01  0.00 | | 0.53  0.22  0.05 | -2.67  -16.31  -2.85 | -24.87  1.01  -3.66 | | -146.73  -22.32  -34.44 |
| Csa03g001630,1  Csa14g001560,1  Csa17g002080,1 | | | AT1G01610 | glycerol-3-phosphate acyltransferase GPAT4 | | | 1.28  1.09  2.10 | 3.43  2.83  4.84 | | 2.37  3.90  3.50 | 2.10  2.15  1.77 | -2.15  -3.01  -1.79 | | 1.04  -1.35  1.06 |
| Csa19g015530.1  Csa01g012370.1  Csa01g012490.1 | | | AT3G11430 | glycerol-3-phosphate acyltransferase GPAT5 | | | 0.33  0.16  0.61 | 1.21  0.00  1.74 | | 0.00  0.00  0.00 | 3.56  NA  2.58 | NA  NA  NA | | NA  NA  NA |
| Csa04g051790.1  Csa05g015440.1  Csa06g040850.1 | | | AT2G38110 | glycerol-3-phosphate acyltransferase GPAT6 | | | 0.62  0.49  0.97 | 3.45  2.32  3.46 | | 5.27  5.43  6.19 | 3.71  3.70  2.60 | -5.26  -6.57  -3.90 | | -1.35  -1.76  -1.44 |
| Csa08g058450.1  Csa13g008080.1  Csa20g008300.1 | | | AT5G06090 | glycerol-3-phosphate acyltransferase GPAT7 | | | 0.00  0.03  0.01 | 0.03  0.09  0.12 | | 0.00  0.00  0.00 | NA  9.58  24.62 | NA  NA  NA | | NA  NA  NA |
| Csa02g001460.1  Csa08g001490.1  Csa13g057000.1 | | | AT4G00400 | glycerol-3-phosphate acyltransferase GPAT8 | | | 0.03  0.01  0.10 | 0.04  0.02  0.27 | | 1.68  4.19  1.90 | 2.71  6.66  3.45 | -82.60  -478.70  -19.21 | | -55.81  -216.30  -5.78 |
| Csa02g067560.1  Csa11g094450.1  Csa18g033950.1 | | | AT5G60620 | glycerol-3-phosphate acyltransferase GPAT9 | | | 12.00  14.70  12.86 | 10.50  12.07  13.83 | | 6.38  3.78  5.08 | 1.02  -1.00  1.10 | 1.18  1.57  1.33 | | 1.24  1.62  1.52 |
| Csa14g015560.1  Csa03g016400.1  Csa17g016850.1 | | | AT1G12640 | lysophosphatidylcholine acyltransferase LPCAT1 | | | 12.89  5.93  8.76 | 11.63  6.15  6.32 | | 8.53  2.64  2.95 | 1.03  1.12  -1.05 | 1.11  1.19  1.37 | | 1.17  1.38  1.33 |
| Csa09g097760.1  Csa07g064380.1  Csa16g055780.1 | | | [AT1G63050.1](http://arabidopsis.org/servlets/TairObject?type=gene&id=29314) | lysophosphatidylcholine acyltransferase LPCAT2 | | | 9.20  13.03  12.62 | 6.10  9.27  8.55 | | 3.32  3.60  4.37 | -1.08  -1.05  -1.07 | 1.34  1.52  1.39 | | 1.23  1.46  1.30 |
| Csa11g012790.2  Csa10g011940.2  Csa12g016580.1 | | | AT4G30580.1 | lysophosphatidyl acyltransferase LPAT1 | | | 3.19  4.38  5.29 | 2.53  2.96  4.03 | | 11.99  15.61  20.29 | 1.01  -1.08  -1.02 | -2.09  -1.86  -1.80 | | -2.19  -2.14  -1.91 |
| Csa06g033410.1  Csa06g033390.1  Csa04g044150.1 | | | AT3G57650 | lysophosphatidyl acyltransferase LPAT2 | | | 1.30  1.26  6.11 | 0.98  1.13  3.95 | | 0.95  0.70  1.65 | -1.00  1.11  -1.10 | -1.32  -1.09  1.50 | | -1.34  1.02  1.37 |
| Csa17g090010.1  Csa03g058140.1  Csa14g059560.1 | | | AT1G51260 | lysophosphatidyl acyltransferase LPAT3 | | | 2.65  1.81  1.79 | 1.69  1.41  1.11 | | 5.34  4.62  2.37 | -1.13  1.00  -1.15 | -1.81  -2.21  -1.69 | | -2.17  -2.32  -2.05 |
| Csa07g046450.1  Csa09g079600.1  Csa16g038920.1 | | | AT1G75020 | lysophosphatidyl acyltransferase LPAT4 | | | 0.71  1.24  0.88 | 1.09  1.19  0.81 | | 2.64  1.37  2.87 | 1.70  1.18  1.17 | -3.57  -1.67  -3.13 | | -2.19  -1.45  -2.86 |
| Csa15g026520.1  Csa01g021830.1  Csa19g026290.1 | | | AT3G18850 | lysophosphatidyl acyltransferase LPAT5 | | | 2.69  1.94  2.88 | 1.58  0.48  0.76 | | 0.89  0.40  0.84 | -1.18  -2.23  -2.00 | 1.33  1.87  1.44 | | 1.12  -1.24  -1.48 |
| Csa01g042590.1  Csa19g056370.1  Csa01g042590.1 | | | AT2G19450 | Acyl-CoA:diacylglycerol acyltransferase DGAT1 | | | 17.57  15.29  17.57 | 16.59  13.51  16.59 | | 1.19  0.62  1.19 | 1.04  1.02  1.04 | 2.94  4.18  2.94 | | 3.34  4.74  3.34 |
| Csa04g037310.1  Csa06g025650.1  Csa09g058550.1 | | | AT3G51520 | acyl-CoA:diacylglycerol acyltransferase DGAT2 | | | 9.43  14.96  8.53 | 9.35  13.49  9.22 | | 2.86  5.13  4.96 | 1.08  1.03  1.12 | 1.44  1.41  1.10 | | 1.63  1.50  1.28 |
| Csa13g016300.1  Csa08g005560.1  Csa20g019000.1 | | | AT5G13640 | phospholipid:diacylglycerol acyltransferase PDAT1 | | | 3.00  1.26  1.91 | 1.64  0.84  1.28 | | 5.74  2.96  3.60 | -1.23  -1.10  -1.10 | -1.71  -2.36  -1.94 | | -2.27  -2.80  -2.26 |
| Csa04g024660.1  Csa06g018480.1  Csa09g035780.1 | | | AT3G44830 | Lecithin:cholesterol acyltransferase family protein PDAT2 | | | 12.11  7.28  14.16 | 19.96  9.71  38.30 | | 0.00  0.01  0.00 | 1.26  1.22  1.43 | NA  133.34  NA | | NA  NA  252.78 |
| Csa19g022610.1  Csa01g018440.1  Csa15g020460.1 | | | AT3G15820.1 | phosphatidylcholine:diacylglycerol cholinephosphotransferase PDCT  (ROD1) | | | 9.00  4.90  6.79 | 13.33  5.63  10.73 | | 2.25  0.88  1.01 | 1.25  1.18  1.30 | 1.56  1.92  2.10 | | 2.14  2.47  3.10 |
| Csa01g010330.1  Csa19g013020.1  Csa15g010950.1 | | | AT3G09560 | phosphatidic acid phosphohydrolase PAH 1 | | | 7.32  7.18  8.00 | 7.79  9.85  11.37 | | 1.03  0.90  1.26 | 1.12  1.23  1.24 | 2.14  2.30  2.00 | | 2.61  3.19  2.77 |
| Csa18g011070.1  Csa20g066660.1  Csa11g072150.1 | | | AT5G42870.1 | phosphatidic acid phosphohydrolase PAH 2 | | | 6.44  5.81  7.87 | 5.51  6.10  7.55 | | 1.25  1.15  1.27 | 1.03  1.13  1.07 | 1.79  1.79  1.98 | | 1.93  2.16  2.27 |
| Csa09g001150.1  Csa06g001110.1  Csa04g002120.1 | | | AT2G01180 | phosphatidate phosphatase LPP1 | | | 0.05  0.00  0.01 | 0.03  0.02  0.00 | | 0.15  0.44  0.05 | 1.16  NA  NA | -7.80  NA  -12.18 | | -11.20  -32.79  NA |
| Csa14g018220.1  Csa03g018880.1  Csa17g020640.1 | | | AT1G15080 | phosphatidate phosphatase LPP2 | | | 1.09  3.01  1.34 | 0.87  1.22  0.36 | | 3.03  3.57  1.53 | 1.04  -1.49  -2.17 | -2.71  -1.43  -1.66 | | -2.78  -2.32  -3.96 |
| Csa17g018860.1  Csa03g017310.1  Csa14g016490.1 | | | AT1G13560.1 | Choline/ethanolaminephosphotransferase AAPT1 | | | 10.32  15.43  11.15 | 7.59  14.63  8.43 | | 3.64  8.79  6.57 | -1.03  1.04  -1.02 | 1.36  1.19  1.13 | | 1.32  1.27  1.10 |
| Csa12g028090.1  Csa11g019460.1  Csa10g017840.1 | | | AT4G25140.1 | Oleosin Ole1 | | | 1081.58  886.59  1284.16 | 2945.32  2357.80  3488.31 | | 0.00  0.00  0.00 | 1.11  1.11  1.11 | NA  NA  NA | | NA  NA  NA |
| Csa11g057650.1  Csa10g047190.1  Csa09048s010.1 | | | AT5G40420.1 | Oleosin Ole2 | | | 347.98  259.07  42.31 | 1247.51  824.93  136.29 | | 0.00  0.00  0.00 | 1.17  1.17  1.35 | NA  NA  NA | | NA  NA  NA |
| Csa04g015780.1  Csa09g014800.1  Csa06g008780.1 | | | AT3G27660.1 | Oleosin Ole4 | | | 214.47  189.91  191.30 | 1235.83  1041.48  1106.17 | | 0.01  0.00  0.27 | 1.31  1.30  1.31 | 244.86  NA  16.31 | | 405.52  NA  24.05 |
| Triacylglycerol Degradation | | | | | | | | | | | | | | |
| Csa11g032860.1  Csa12g049890.1  Csa10g028670.1 | | | At4g16820.1 | DAD1-LIKE LIPASE 1 | | | 0.08  0.10  0.03 | 0.00  0.07  0.00 | | 0.00  0.00  0.01 | NA  1.10  NA | NA  NA  -1.71 | | NA  NA  NA |
| Csa05g004460.1  Csa06g051180.1 | | | [AT2G44810.1](http://arabidopsis.org/servlets/TairObject?type=gene&id=434685) | Defective Anther Dehiscence DAD1 | | | 0.09  0.18 | 0.03  0.03 | | 0.00  0.00 | -1.65  -3.65 | NA  NA | | NA  NA |
| Csa15g059780.1  Csa01g038410.1  Csa19g040900.1 | | | AT2G06925.1 | Phospholipase A2-Alpha  PLA2-Alpha | | | 34.07  6.02  21.89 | 22.56  3.55  23.55 | | 2.28  1.45  6.73 | -1.06  -1.15  1.08 | 2.64  1.58  1.48 | | 2.60  1.37  1.67 |
| Csa19g046330.1  Csa15g071200.1  Csa01g035770.1 | | | At2g15230.1 | Triacylglycerol Lipase Lip 1 | | | 1.40  7.06  2.38 | 1.63  8.38  3.52 | | 3.38  4.43  7.99 | 1.31  1.17  1.41 | -2.36  1.05  -2.22 | | -1.84  1.28  -1.55 |
| Csa13g006100.1  Csa08g060480.1  Csa20g005210.1 | | | AT5G04040.1 | SUGAR-DEPENDENT1 SDP1 | | | 2.56  0.69  2.10 | 2.42  0.77  2.14 | | 0.84  0.28  0.41 | 1.11  1.37  1.17 | 1.33  1.16  1.95 | | 1.53  1.63  2.46 |
| Csa04g043530.1  Csa09g069610.1  Csa06g032880.1 | | | AT3G57140.1 | sugar-dependent 1-like SDPL1 | | | 0.62  0.23  0.45 | 0.45  0.20  0.34 | | 0.11  0.12  0.08 | -1.01  1.19  1.05 | 2.33  -1.10  2.27 | | 2.38  1.08  2.49 |
| Csa17g092830.1  Csa14g062020.1  Csa03g059760.1 | | [AT1G52760.1](http://arabidopsis.org/servlets/TairObject?type=gene&id=28725) | | Lysophospholipase 2 MAGT | | | 152.67  66.71  40.97 | 158.04  89.23  62.92 | | 9.56  9.90  9.75 | 1.03  1.11  1.16 | 2.28  1.85  1.63 | | 2.43  2.13  1.97 |
| The relative expression abundance of the selected genes associated with fatty acid, TAG synthesis and degradation are shown. The original RPKM values are shown here to estimate the level of gene expression, while the Log2 transformed RPKM values were used to estimate the fold changes in gene expression and those values were used in Figure 6. | | | | | | | | | | | | | | |

| Table S10. Comparative Quantification of transcript levels | | | | | | | |
| --- | --- | --- | --- | --- | --- | --- | --- |
|  | | **Cs-14 Vs Cs-21** | | **Cs-14 Vs Cs-Leaf** | | **Cs-21 Vs Cs-Leaf** | |
| ID | **Gene name** | **S** | **Q** | **S** | **Q** | **S** | **Q** |
| Csa02g067560.1  Csa11g094450.1  Csa18g033950.1 | **GPAT9 glycerol-3-phosphate acyltransferase 9** | **1.02**  **-1.00**  **1.09** | **-1.46** | **1.18**  **1.57**  **1.32** | **8.92** | **1.23**  **1.61**  **1.52** | **6.02** |
| Csa06g033410.1  Csa06g033390.1  Csa06g033430.1 | **LPAT2 lysophosphatidyl acyltransferase 2** | **-1.00**  **1.11**  **-1.05** | **-0.36** | **-1.31**  **-1.09**  **1.13** | **2.90** | **-1.33**  **1.02**  **1.07** | **2.17** |
| Csa09g001150.1  Csa06g001110.1  Csa04g002120.1 | **LPP1 phosphatidic acid phosphatase 1** | **1.15**  **1.00**  **1.00** | **1.47** | **-7.79**  **NA (LS)**  **-12.11** | **-2.40** | **-11.19**  **-32.79**  **NA (LS)** | **-1.31** |
| Csa14g018220.1  Csa03g018880.1  Csa17g020640.1 | **LPP2 phosphatidic acid phosphatase 2** | **1.04**  **-1.49**  **-2.16** | **-1.43** | **-2.71**  **-1.42**  **-1.66** | **2.35** | **-2.77**  **-2.32**  **-3.96** | **0.54** |
| Csa01g042590.1  Csa19g056370.2  Csa15g084220.1 | **DGAT1 membrane bound O-acyl transferase (MBOAT) family protein** | **1.04**  **1.02**  **1.13** | **1.07** | **2.94**  **4.18**  **2.56** | **4.37** | **3.33**  **4.74**  **3.21** | **5.42** |
| Csa04g037310.1  Csa09g058550.1  Csa06g025650.1 | **DGAT2 diacylglycerol acyltransferase family** | **1.07**  **1.11**  **1.02** | **1.48** | **1.44**  **1.10**  **1.41** | **2.22** | **1.63**  **1.27**  **1.50** | **2.84** |
| Csa13g016300.1  Csa08g005560.1  Csa20g019000.1 | **PDAT1 phospholipid:diacylglycerol acyltransferase** | **-1.23**  **-1.10**  **-1.09** | **-0.29** | **-1.71**  **-2.36**  **-1.93** | **-0.24** | **-2.27**  **-2.79**  **-2.25** | **-0.88** |
| [Csa12g028090.1](http://camelinadb.ca/prairiegold/cgi-bin/do_blast.cgi#34889)  [Csa11g019460.1](http://camelinadb.ca/prairiegold/cgi-bin/do_blast.cgi#5956)  [Csa10g017840.1](http://camelinadb.ca/prairiegold/cgi-bin/do_blast.cgi#69354) | **Oleosin 1** | **1.10**  **1.10**  **1.11** | **1.48** | **NA (SS)**  **NA (SS)**  **NA (SS)** | **8.57** | **NA (SS)**  **NA (SS)**  **NA (SS)** | **10.03** |
| Csa04g015780.1  Csa09g014800.1  Csa06g008780.1 | **Oleosin 4** | **1.30**  **1.29**  **1.31** | **1.75** | **244.8**  **NA(SS)**  **16.3** | **15.66** | **405.51**  **NA (SS)**  **24.05** | **17.39** |
| Csa06g028810.1  Csa04g040400.1  Csa09g064030.1 | **WRl1 Integrase-type DNA-binding superfamily protein** | **-1.11**  **-1.13**  **-1.08** | **-0.14** | **9.17**  **15.83**  **11.56** | **6.94** | **9.34**  **16.13**  **12.27** | **6.81** |
| Csa17g092850.1  Csa03g059790.1  Csa17g092830.1 | **MGAT1 lysophospholipase 2** | **1.75**  **1.43**  **1.03** | **1.03** | **1.30**  **1.24**  **2.27** | **3.98** | **2.62**  **1.88**  **2.42** | **5.03** |
| Csa19g022610.1  Csa15g020460.1  Csa01g018440.1 | **PDCT phosphatidic acid phosphatase-related / PAP2-related** | 1.24  1.30  1.18 | **1.16** | **1.56**  **2.09**  **2.26** | **4.29** | **2.14**  **3.10**  **2.46** | **4.81** |
| Csa11g050970.1  Csa10g040780.1  Csa12g074630.1 | **WSD1 O-acyltransferase (WSD1-like) family protein** | **1.00**  **4.88**  **1.00** | **1.52** | **NA(LS)**  **-21.01**  **NA (LS)** | **-2.47** | **NA (LS)**  **-4.57**  **NA (LS)** | **-0.93** |
| The relative gene expression analysis for the thirteen selected candidate genes associated with lipid metabolisms measured by using both RNA-Seq and qRT-PCR approach. Fold change in log2 scale was used to represent the relative expression. *S* for RNA-Seq data and *Q* for qRT-PCR data. The three values for gene expression measurements obtained from the RNA-Seq analysis represent the data from the three genomic copies, while there is only one value for qRT-PCR data which represent the average of the three biological replicates of each gene by using PCR primers designed to amplify the conserved regions of the three genomic copies. Cs-14 and Cs-21 are the camelina seeds at 10-15 and 16-21 days after flowering DAF, respectively, while Cs-Leaf is Camelina leaf. The negative values in fold changes indicates the direction of change, NA indicates that fold change could not be calculated, because the value is 0 of any compared pair, that when divided by 0, it gives NA. In Cs-14 Vs Cs-21 comparisons, Cs-14 was used as a reference, while in Cs-14 Vs Cs-Leaf and Cs-21 Vs Cs-Leaf comparisons, Cs-Leaf was used as a reference. NA: the fold change calculation is not applicable; SS: seed specific; LS: Leaf specific | | | | | | | |

| Table S11. The relative expression of the key genes in TAG biosynthesis in developing seeds of Camelina | | |
| --- | --- | --- |
| *Gene name | **Gene Expression (relative to β-actin)** | |
|  | **Cs-14** | **Cs-21** |
| WRl1 | 2.31 | 2.36 |
| GPAT1 | 0.05 | 0.09 |
| GPAT2 | 0.15 | 0.11 |
| GPAT3 | 0.01 | 0.00 |
| GPAT4 | 0.08 | 0.35 |
| GPAT5 | 0.02 | 0.09 |
| GPAT6 | 0.04 | 0.29 |
| GPAT7 | 0.00 | 0.01 |
| GPAT8 | 0.00 | 0.09 |
| GPAT9 | 0.70 | 1.14 |
| LPCAT1 | 0.49 | 0.76 |
| LPCAT2 | 0.62 | 0.75 |
| LPAT1 | 0.23 | 0.30 |
| LPAT2 | 0.15 | 0.19 |
| LPAT3 | 0.11 | 0.13 |
| LPAT4 | 0.05 | 0.10 |
| LPAT5 | 0.13 | 0.09 |
| DGAT1 | 0.89 | 1.47 |
| DGAT2 | 0.58 | 1.01 |
| MGAT | 4.61 | 10.34 |
| PDAT1 | 0.11 | 0.12 |
| PDAT2 | 0.59 | 2.14 |
| PDCT | 0.37 | 0.93 |
| PAH1 | 0.40 | 0.91 |
| PAH2 | 0.36 | 0.60 |
| Ole1 | 57.59 | 276.32 |
| Ole2 | 11.50 | 69.42 |
| Ole4 | 10.55 | 106.35 |
| SDP1 | 0.09 | 0.17 |
| SDP1L | 0.02 | 0.04 |
| NHO1 | 0.23 | 0.25 |
| ATS1 | 0.16 | 0.10 |
| Caleosins | 2.28 | 3.64 |
| DGK | 0.17 | 0.16 |
| PLD | 0.74 | 0.95 |
| PLA2 | 1.10 | 1.56 |
| AAPT | 0.65 | 0.96 |
| The gene expression is normalized to the expression of the housekeeping gene β-Actin. The values represented the mean expression of the three genomic copies for each gene divided by the mean expression of the three genomic copies of β-Actin. Cs-14, 10-15 days after flowering; Cs-21, 16-21 days after flowering. | | |
